# Between similarity and difference: network dynamics of the hippocampal- parahippocampal circuitry in pattern separation of male Wistar rats

**DOI:** 10.3389/fncel.2025.1648536

**Published:** 2025-11-18

**Authors:** Ana Paula de Castro Araujo, Jeanderson Soares Parente, Sofia Lucena de Oliveira Coutinho, Rochele Castelo-Branco, Ywlliane S. R. Meurer, Flávio Freitas Barbosa

**Affiliations:** Memory and Cognition Studies Laboratory, Department of Psychology, Federal University of Paraíba, João Pessoa, Brazil

**Keywords:** object recognition memory, pattern separation, hippocampal network, graph analysis, dentate gyrus, PV cells

## Abstract

**Introduction:**

Studies indicate that pattern separation for spatial and object information involves structures of the temporal cortex (lateral entorhinal and perirhinal cortices) and hippocampus (dentate gyrus and CA3), which are particularly sensitive to aging. However, little is known about how the hippocampal network, the anteroposterior axis of these regions, and the excitatory-inhibitory circuit contribute to the recognition and separation of object patterns.

**Methods:**

This study investigated the expression of c-Fos and PV along the anteroposterior axis of the hippocampus in a multi-trial task to assess the recognition of novel objects and recognition of novel objects with different levels of similarity. Five groups of animals performed tasks with different similarity demands (NOR, DIST, 25, 50, 75%).

**Results:**

The data showed that conditions of greater similarity led to increased c-Fos expression in CA3c and Hilus in the rostral hippocampus. Graph analysis revealed that hippocampal networks became more densely interconnected and efficient as object similarity increased. Furthermore, different patterns of cluster organization emerged depending on task demands. Besides, the granule cell layer along the dorsoventral axis exhibited greater activation of inhibitory neurons (PV+/c-Fos+) under conditions of higher similarity. Differential inhibitory/excitatory control of the DG-CA3 microcircuit network is seen across conditions. Modeling the DG layers revealed robust control of GCs through direct and indirect effects of interneurons present in the hilus and granule layer. Bidirectional direct and indirect effects of MCs on GCs were observed.

**Discussion:**

These results contribute to our understanding of how brain networks and DG excitatory/inhibitory microcircuits are jointly engaged in object recognition memory and disambiguation of overlapping inputs.

## 1 Introduction

Episodic memory refers to the recollection of unique and specific events, and the ability to distinguish between similar experiences is considered essential to avoid interference between such representations. Thus, beyond mere recognition, it is believed that the brain generates distinct representations of similar events—less overlapping and capable of being associated with different outcomes—through a process known as pattern separation, which is fundamental for the successful storage of episodic memories (Josey and Brigman, 2015; Gilbert and Kesner, 2006; Miranda et al., 2017).

Recent efforts have focused on developing specific protocols to investigate pattern separation in rodents. By manipulating the degree of feature overlap between objects, various studies have evaluated the recognition of similar items through different approaches, such as mazes or open-field environments, and protocols based on either choice or free exploration—always systematically controlling object similarity (Johnson et al., 2017; Miranda et al., 2017; Miranda et al., 2021; Clark et al., 2011; Norman and Eacott, 2004). More recent findings highlight the hippocampus—particularly the dentate gyrus (DG)—as a central region in the pattern separation process. Anatomical, physiological, behavioral, and lesion-based evidence supports the hypothesis that the DG–CA3 circuit is involved in pattern separation and completion, respectively, based on inputs from the entorhinal cortex to the hippocampus (Senzai, 2018; Kang and Toyoizumi, 2024; Leutgeb et al., 2007; GoodSmith et al., 2019; Neves et al., 2022; Hunsaker and Kesner, 2008).

Studies have demonstrated that inhibitory activity is essential for the successful encoding and retrieval of memory (Johnson et al., 2019; GoodSmith et al., 2019; Senzai, 2018; Kang and Toyoizumi, 2024). The tight inhibitory control exerted by interneuron populations within the DG, along with the anatomical organization of projections from the entorhinal cortex, results in relatively sparse activity in this region (Johnson et al., 2019; Rolls, 2013; Guzman et al., 2016; Senzai, 2018). This sparse activation is fundamental to the original proposal that the encoding of distinct memories occurs through orthogonalization within the DG (Rolls et al., 1998; Kassab and Alexandre, 2018). The cellular composition of the DG provides a functional basis for pattern separation: mossy cells, which are more responsive and active, can modulate the activity of granule cells—typically more silent and hypoactive—through mechanisms of indirect inhibition or direct excitation (Bernstein et al., 2019; GoodSmith et al., 2019). In this way, mossy cells may integrate circuits specialized in detecting and encoding distinct (non-overlapping) information, while granule cells may be more involved in encoding highly similar input (Morales et al., 2020).

The sparse activity in the DG, combined with the associative network of CA3, facilitates the retrieval of complete patterns from partial cues, thereby contributing to pattern completion (Brunel, 2016). Moreover, there appears to be a functional specialization along the longitudinal axis of CA3, with distinct roles in pattern separation and pattern completion (Lee et al., 2022; Berdugo-Vega et al., 2023).

Despite these advances, many questions remain open regarding the underlying mechanisms of this process, and few studies have investigated object-based pattern separation (Johnson et al., 2021; Miranda et al., 2021). To the best of our knowledge, no study has yet explored how the functionally and connectively heterogeneous subregions of the hippocampus contribute to this process (Cooper et al., 2022; Sugar et al., 2011; Sethumadhavan et al., 2022). Most studies focus primarily on differential activation of regions (e.g., immediate early gene expression) across conditions, without examining network dynamics within hippocampal and parahippocampal regions involved in object recognition and pattern separation (Castelo-Branco et al., 2025). Furthermore, the interaction between inhibitory and excitatory networks during object recognition and pattern separation tasks remains poorly understood (Johnson et al., 2021; Kassab and Alexandre, 2018).

We hypothesize that c-Fos expression varies across hippocampal subregions depending on the level of similarity between stimuli, reflecting changes in neural network dynamics according to the experimental condition. Specifically, we propose that under high similarity, there is increased recruitment of parvalbumin (PV) interneurons within the DG–CA3 circuit, and that this inhibitory network plays a crucial role in modulating the response of granule cells. To test this, we employed a novel object recognition (NOR) task and a similar novel object recognition (SNOR) task based on the protocol proposed by Johnson et al. (2017). We measured c-Fos expression, alone or co-labeled with PV, to assess neuronal activity along the anteroposterior and dorsoventral axes of the hippocampus, as well as in parahippocampal regions, since both are involved in object recognition and pattern separation (Miranda et al., 2021; Bernstein et al., 2019; David et al., 2020). Using graph theory analysis and Bayesian structural equation modeling, we identified broader and more interconnected brain networks under conditions of high similarity, along with a robustly engaged network modulating granule cells (GCs) in those conditions.

## 2 Materials and methods

### 2.1 Animals

Fifty male Wistar rats (13 ± 2 weeks old) were used. The animals were housed individually in polyethylene cages (30 cm long × 20 cm wide × 19 cm high). All animals were provided by the Prof. Eduardo Barbosa Beserra Animal Facility at the State University of Paraíba (UEPB). All procedures were conducted in accordance with Brazilian legislation for the use of animals in research (Arouca Law No. 11.794/08), with every effort made to minimize stress, pain, and discomfort (CEUA protocol number: 5276130921).

### 2.2 Experimental protocol

#### 2.2.1 Experimental apparatus

**FIGURE 1:**
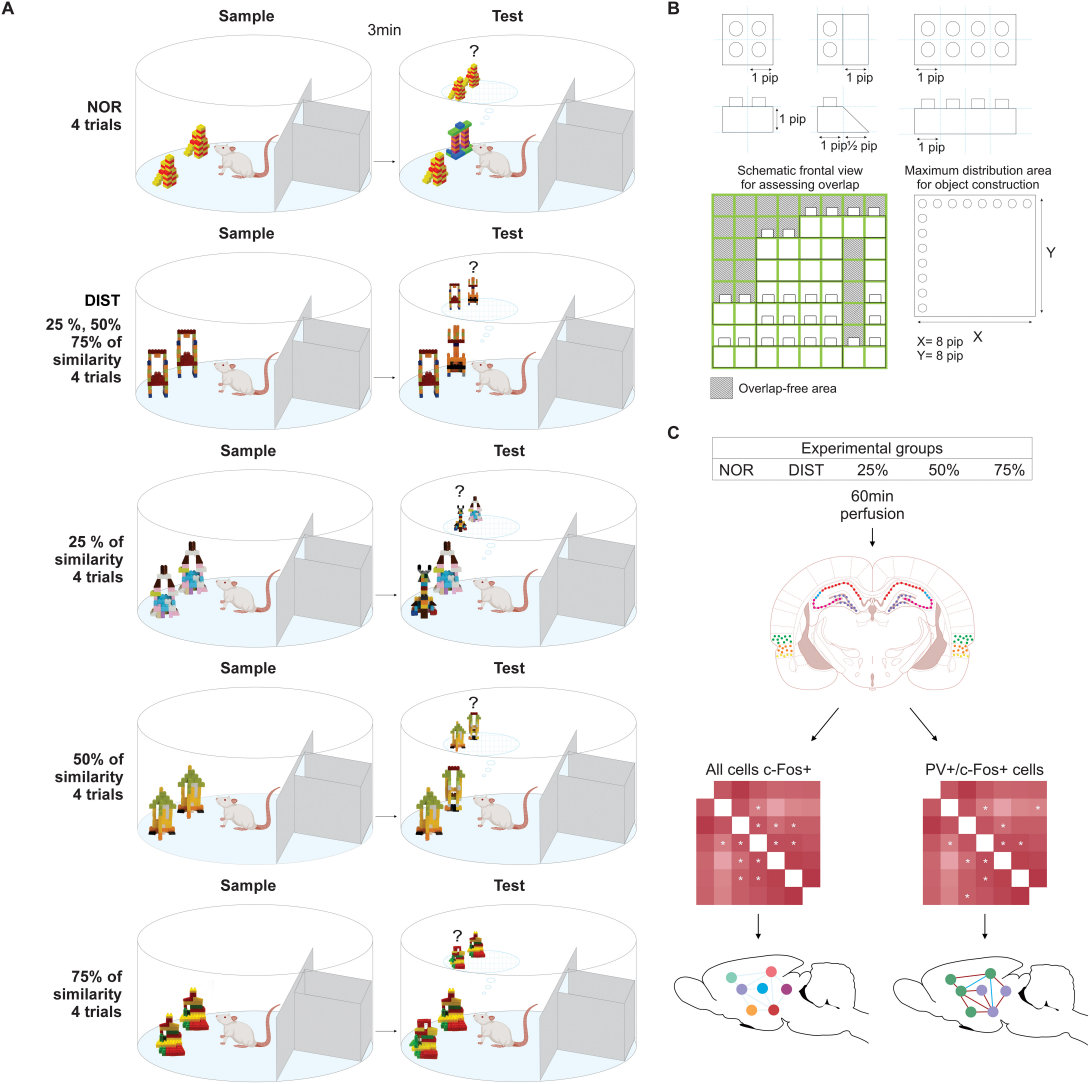
Experimental design for behavioral and immunofluorescence data. **(A)** Rats performed the novel object recognition (NOR) and similar novel object recognition (SNOR) tasks with multiple trials under different conditions: all similarity levels grouped (25, 50, and 75%) or separated by similarity level. **(B)** Representative schematic of object overlap calculation using Lego blocks, adapted from Johnson et al. (2017). **(C)** Assessment of neural areas recruited in each condition via c-Fos expression and PV+/c-Fos+ double labeling, followed by the construction of two interregional correlation matrices: all c-Fos+ cells (left) and activated PV+/c-Fos+ and c-Fos+ only cells (right).

The experimental apparatus consisted of an open field 90 cm in diameter, with walls 45 cm high. A black opaque box (30 cm long × 15 cm wide × 40 cm high) was attached to the internal side of the field, with a manually operated door connecting it to the open area, controlled from an external room. The field was divided using an acrylic wall and covered with a black tarp to reduce distal or proximal cues (see Figure 1A).

#### 2.2.2 Handling, habituation, and shaping

Animals were placed in the testing room 30 min prior to any behavioral procedure for acclimatization. Before testing began, animals were handled by the experimenter for 15 min over 5 consecutive days to reduce stress and increase familiarity.

Animals underwent 23 h of water deprivation prior to testing to increase motivation and exploratory drive (Polo-Castillo et al., 2019). During shaping, animals were trained to exit and re-enter the box within a 3-min interval. Drops of water scattered in the field and the box motivated the rats’ movements, and these drops were gradually reduced throughout training. A pair of drops was always placed in front of the future object locations. During training, rats were exposed to objects with drops placed in front of them, but different objects were used during testing. Rats were considered shaped once they exited and re-entered the box three consecutive times within the correct 5-min interval.

#### 2.2.3 Objects

Objects used in all tasks were built from LEGO^®^ bricks. For the NOR task, objects with different patterns were constructed. For the SNOR task, objects were created with varying levels of similarity. Object pairs were matched in terms of general volume, shape, and texture, but systematically varied in the number of shared visual features, based on the objects developed by Johnson et al. (2017), measuring approximately 6–9 cm in height on a 6.5 × 6.5 cm LEGO base. Systematic similarity levels (∼25, ∼50, ∼75%) were defined based on volume and visible feature overlap. Similarity was calculated according to the protocol by Johnson et al. (2017). First, the object’s maximum 3D volume was determined by multiplying its height (Z), width (X), and depth (Y) by the number of “pips” on each axis. This yielded the total 3D volume. One “pip” corresponds to one LEGO 3D block unit. The total pip count of an object was calculated, and then the pip quantity corresponding to 25, 50, or 75% of that total was determined. New objects were then constructed to control the degree of pip overlap according to the desired percentage (see Figure 1B). Overlap was also assessed based on shared surface features visible on the front face of each object (Johnson et al., 2017; de Castro Araujo et al., 2025; see Figure 1A for examples of objects).

#### 2.2.4 Experimental design

Animals were divided into five groups: 1—NOR; DIST (Distributed similarity levels); and individual 25, 50, and 75% similarity groups. The NOR group completed one block of four trials for the NOR task. The DIST group performed the SNOR task with a randomized and counterbalanced sequence of similarity levels (25, 50, and 75%) across four trials. The inclusion of the DIST group aimed to simulate a more naturalistic scenario, where memories with varying degrees of similarity compete simultaneously. By combining different levels of interference (25, 50, and 75%), this group allows us to test whether the neural responses seen in isolated conditions are additive, synergistic, or non-linear when experienced together. This approach helps assess the robustness and interaction of pattern separation mechanisms under more complex and ecologically valid conditions.

The remaining three groups each underwent the SNOR task with a fixed similarity level (25, 50, or 75%), across a block of four trials (see Figure 1A). Only animals with a D2 discrimination index above chance (> 0) were included in the immunofluorescence analyses. Twenty-four animals were excluded based on this criterion (NOR = 2, DIST = 3, 25% = 3, 50% = 4, 75% = 4), leaving 36 animals for the analyses (NOR = 8, DIST = 8, 25% = 7, 50% = 7, 75% = 6).

#### 2.2.5 Novel object recognition task

The novel object recognition (NOR) task consisted of two phases: a sample and a test phase, each lasting 3 min, with a 3-min interval between them. We selected this interval as an intermediate duration, falling between the shorter intervals typically used in multiple-trial paradigms and the longer intervals employed in classical NOR tasks. Our previous studies, which involved more trials and extended object exposure, showed that a 3-min interval yielded robust discrimination performance while maintaining the animals’ motivation to explore the objects throughout the trials. Four trials were conducted, each separated by a 5-min interval (Araujo et al., 2021; de Castro Araujo et al., 2025; Ameen-Ali et al., 2012). This continuous approach, as described by Ameen-Ali et al. (2012), allows the collection of a significantly larger number of trials per animal within a single testing session, maintaining the same statistical power as the traditional approach, which requires one session for each animal. Thus, in addition to considerably reducing the number of animals used, this strategy also enables different interventions across trials (Chan et al., 2018; Seel et al., 2018). At the beginning of the sample phase, the animal was placed inside the central box; the door was opened, the rat exited, and the door was then closed, marking the start of the sample phase. In this phase, the animal was exposed to two identical LEGO objects. At the end of the sample phase, the door was reopened and the animal returned to the box. After the predetermined interval, the door was opened again, initiating the test phase. In the test, the rat was presented with an identical copy of one of the sample objects and a completely distinct new LEGO object. All objects were placed in the same positions as in the sample phase, as illustrated in Figure 1A, the distance between objects was set at 15 ± 1 cm. The objects, as well as the field, were cleaned with a 70% alcohol solution at the end of each experimental day and with a 5% alcohol solution between sessions to prevent olfactory cues. The positions and shapes of the LEGO objects were counterbalanced for the animals in this and the subsequent task.

#### 2.2.6 Similar novel object recognition task

The similar novel object recognition (SNOR) task followed the same phases, intervals, and number of trials as the novel object recognition (NOR) task. However, in the test phase, one of the objects from the sample phase was replaced with a novel object that had a specific degree of similarity: 25, 50, or 75%. For the DIST, the degree of similarity was changed in each test and randomized across trials. For the 25, 50, and 75% groups, the similarity level was kept constant across trials (see Figure 1A). For all groups, new sets of objects were used for each trial.

#### 2.2.7 Perfusion and immunofluorescence processing for c-Fos and PV

The perfusion was performed 1 h after completing the behavioral procedures. The animals were anesthetized with sodium thiopental (50 mg/kg, i.p.) and transcardially perfused with 200 mL of heparinized saline solution (0.9% NaCl), followed by 200 mL of cold 4% paraformaldehyde (PFA) fixative in phosphate buffer solution (PBS, 0.1 M), until ready for brain removal. The brains were post-fixed in 4% PFA plus 0.1 M PBS with 20% sucrose for 3 days at 4°C before being embedded in a cryoprotectant solution (PBS with 30% sucrose) for subsequent coronal plane of sectioning using a cryostat (Leica, Germany), with each section being 50 μm thick for analysis of the dorsal hippocampus and adjacent areas (Castelo-Branco et al., 2025). All sections were stored in an antifreeze solution. Initially, sections were placed in blocking solution (5% Molico^®^ milk in 0.1 M PBS) for 1 h. Floating sections were then incubated for 18 h with two specific primary antibodies: rabbit anti-c-Fos (Cell Signaling, MA, United States; 1:3,000) and goat anti-PV (Sigma-Aldrich, 1:3,000), diluted in 0.1 M PBS, pH 7.4, plus 0.03% Triton X-100 (ICN Biomedicals). All antibody dilutions were previously tested. Sections were then incubated separately with fluorescent secondary antibodies. First, Alexa 594 donkey anti-goat (Abcam-Cambridge, 1:500) for PV, diluted in 0.3% Triton X-100, was applied for 90 min. Next, Alexa 488 donkey anti-rabbit (Abcam-Cambridge, 1:500) for c-Fos was applied.

Sections were then immersed in phosphate buffer (PB) 0.1 M, pH 7.4, containing 2% DAPI, and mounted on gelatin-coated slides using Fluoroshield mounting medium (Sigma-Aldrich). At the end of the immunofluorescence procedure, dorsal hippocampal sections from one animal from the NOR group, one from the DIST group, and one from the 25% group and ventral hippocampal sections from the NOR group were excluded due issues during tissue processing and not included in the immunofluorescence analysis of this areas, leaving 33 animals for behavioral analysis (NOR = 7, DIST = 7, 25% = 7, 50% = 6, and 75% = 6). The immunofluorescence results were evaluated using a Nikon Eclipse TS100 fluorescence microscope (Tokyo, Japan).

#### 2.2.8 Brain region delimitation

The delimitation of the cytoarchitectonic subfields within the hippocampus and parahippocampus areas for immunofluorescence analysis was based on the morphological criteria described in the literature (Amaral et al., 1987; Scharfman, 2016). Anatomical references included the stereotaxic coordinate atlas (Paxinos and Watson, 2013) as well as digital coronal series showing parvalbumin-labeled cells in the rat hippocampal formation by Kjonigsen et al. (2011) and Boccara et al. (2014). c-Fos immunoreactivity was analyzed across the rostrocaudal and dorsoventral axis of hippocampal formation by evaluating rostral (interaural: 5.28–5.04 mm), medial (interaural: 4.56–4.32 mm) and caudal (interaural: 3.48–3.24 mm) sections. A distinct evaluation of the dorso and ventral areas of the caudal hippocampus was also made, since different studies have pointed out the functional and projection specifications of these regions, therefore dividing them into dorsal (CA1*d*, CA3*d*, Hilus*d*, and GCL*d*) and ventral regions (CA1*v*, CA3*v*, Hilus*v*, and GCL*v*) (Houser et al., 2021; Malhotra et al., 2012; López-Oropeza et al., 2022; Strange et al., 2014; Van Strien et al., 2009; Fanselow and Dong, 2010). Other functional distinctions among the CA3 subfields (CA3a, CA3b, and CA3c) were also taken into account (Lee et al., 2022) as well as the subdivision of the DG into granular cell layer (GCL) and Hilus (polymorphic layer) (Amaral et al., 1987; Houser et al., 2021). For the parahippocampal areas, further segmentation was made considering the functional specialization of the Perirhinal cortex (Prh36 and Prh35) and Lateral Entorhinal cortex (LEC). Each region was divided into superficial (sl) and deep (dl) layers corresponding to afferent (layers II and III) and output (IV to VI) connectivity patterns with the hippocampus: Prh36*sl*, Prh36*dl*, Prh35*sl*, Prh35*dl*, LEC*sl*, and LEC*dl* (see Figure 3A for a better understanding) (Sugar et al., 2011; Sethumadhavan et al., 2022; Van Strien et al., 2009, for representative plates by group, see Supplementary Figure 1).

All criteria for defining and delimiting the regions of interest (ROI) were applied by an experienced experimenter blinded to the experimental condition, in order to minimize interpersonal variability and classification bias. The imaged sections were individually and sequentially merged digitally to compose all regions of interest. To delimitate the granule cell layer and hilus, we overlaid vectorized images from Paxinos atlas (Paxinos and Watson, 2013) corresponding to each analyzed section as a reference, then draw lines were manually traced for each ROI in all sections using the Adobe Photoshop software and following the criteria discussed by others (Amaral et al., 1987; Scharfman, 2012) that define: (1) the GCL as the area containing cell bodies between the hilus and the molecular layer, and (2) the hilus as the area between the two GCL layers in a V-shape, and adjacent to the end of the pyramidal cell layer of the CA3c area. Background analysis within-sections was applied uniformly to all images previously counting cells in order to reduce background and improve the visualization of the brightest cells. To do so, channel dye separation in each image was performed, and the measurement of intensity was performed in three different areas without cells in each section prior to applying the background subtract (standardized radius = 40) based on the “rolling ball” algorithm (Sternberg, 1983; Bootman et al., 2013). All separated channels were analyzed and merged using the appropriate function in the Icy software. As positive cells were manually counted within each ROI, no brightness or contrast optimization, nor any binary image transformations, were applied after background optimization in order to avoid introducing artifacts into the data. Pre-settings measurements of signal intensity were performed by measuring the nucleus and PV cell areas to estimate an acceptable overlap percentage area for accurate cell counting. Also, a specific signal intensity limit for each channel was measured to define the standard minimum signal intensity value of 1.5X compared with background. Cells were considered positive for PV and/or c-Fos if they met the following criteria: (1) typical neuronal nuclear morphology, (2) the brightest fluorescence signal corresponding to at least 1.5X intensity limit measured for each appropriate channel (green for c-Fos, red for PV and blue for DAPI), and (3) co-staining confirmed by the nuclear marker DAPI staining. For double-localization analyses, c-Fos plus DAPI cells were considered double-labeled if the spatial overlap occurred in at least 80% of the entire cell count. For triple co-localization, cells were considered positive if double co-localization (c-Fos/DAPI) occupied at least 25% of the entire area of PV stained cells, in order to minimize artifacts and/or false positives. Counting c-Fos+ cells was performed manually using cluster sampling, with a randomly selected representative subpopulation in the parahippocampal regions. PV+c-Fos+ double-labeled cell counts were restricted to Hilus, GCL, and CA3 regions. To normalize c-Fos expression levels across animals and groups, values were expressed using the formula: (individual value/group mean) * 100.

### 2.3 Graph theoretical analysis

Brain networks were constructed based on all c-Fos-positive cells. A second network was then created by subdividing cell types in PV+/c-Fos+ activated interneurons and c-Fos+ activated cells population. Both networks were built from significant positive and negative Spearman correlations (*p* < 0.05) for each condition, and were quantitatively characterized using theoretical graph metrics. The analysis was performed in RStudio using the “corrplot” package. Each brain area was represented by a node of a certain size (degree), reflecting the number of brain regions co-activated with that node. All significant correlations between brain areas were represented as connections between nodes, with connection thickness proportional to correlation strength.

For graph analyses, the “igraph” package was used in RStudio, with custom routines developed by the Memory and Cognition Studies Laboratory (LEMCOG). One key objective of graph theory is to identify central nodes, thereby revealing their critical role in information flow within the network. For this, three centrality measures were calculated for each node: degree, strength, and betweenness centrality. Node strength corresponds to the sum of all significant covariances in an area. Higher values indicate greater network centrality. Betweenness centrality refers to the number of shortest paths between other nodes that pass through a given node, reflecting the role of a brain region as an intermediary in communication within the network. While degree and strength reflect static node centrality, betweenness centrality indicates the importance of nodes in network communication. To define hub regions, we first examined values for degree, strength, and centrality; nodes within the 90th percentile for these measures were defined as hubs. Regions with high centrality values were also considered hubs. Lastly, we verified whether these values were above chance; only regions with significant values were considered hubs.

Another aim of graph theory is to characterize overall network efficiency. Networks are more efficient when nodes are well connected with short paths between them. In contrast, networks with dispersed connections and longer paths tend to have lower efficiency. More efficient networks may facilitate faster and more effective neural signal transmission (Fornito et al., 2016). Two measures were used to assess efficiency: global clustering coefficient and global efficiency. The global clustering coefficient assesses network segregation based on the probability that two nodes connected to a common neighbor are also connected. Global efficiency reflects the network’s integration and the speed at which information is exchanged across the network, considering the shortest paths between nodes (Rubinov and Sporns, 2010). Cluster analyses were guided by the Louvain algorithm, from which network modularity and modules were extracted (resolution: full c-Fos+ network = 1; PV+/c-Fos+ plus c-Fos+ activated network = 1.3). The resolution value was defined based on the most frequently repeated number of modules during bootstrap sampling.

We also used the mean network strength to assess overall efficiency. For statistical comparison between groups, classical bootstrap analysis was employed. One hundred samples were generated from the original correlation matrix. Random models were calculated by shuffling matrix rows and columns while keeping the main diagonal intact (preserving structural integrity). After generating the random models, bootstrap tests and permutations were performed to compare networks. van Wijk et al. (2010) normalization by random networks and lattices was performed to allow comparison between networks with different densities.

### 2.4 Bayesian structural equation models

A structural equation model (SEM) was estimated through Bayesian inference using c-Fos and PV immunofluorescence data. This analysis was conducted with the *blavaan* package (Merkle and Rosseel, 2018) in RStudio. The estimation was performed via Markov Chain Monte Carlo (MCMC) sampling using the Stan interface, configured by the argument target = “stan.” For each model, 4 independent chains were executed with 1,000 burn -in iterations to ensure the initial convergence of the chains, followed by 5,000 samples for parameter estimation. The convergence of the MCMC chains was assessed using the scaling potential index ($\hat{\text{R}}$), as recommended in the literature (Gelman et al., 2013; Merkle and Rosseel, 2018). $\hat{\text{R}}$ values close to 1 indicated good convergence for all estimated parameters.

The fit of Bayesian models was assessed mainly through the *posterior predictive p- value* (PPP), which indicates how much the observed data are compatible with the data expected by the model. PPP values close to 0.5 suggest a good fit, while very low values (e.g., less than 0.05) may indicate that the model does not represent the data well (Gelman et al., 1996; Scheel et al., 2021). In addition to the PPP, other adjustment criteria widely used in Bayesian models were also used, such as DIC (*Deviance). Information Criterion*; Spiegelhalter et al., 2002), the WAIC (*Watanabe- Akaike* Information Criterion; Watanabe, 2010) and LOOIC (*Leave-One-Out Information Criterion*; Vehtari et al., 2017), which allow comparing the quality of fit between different models, taking into account the complexity of the model and its predictive capacity.

Given the limited sample size, we reduced the complexity of the model and prioritized connections that most directly reflect interactions between major cell types within the dentate gyrus. Two Bayesian models with the same structure were compared in the direct regressions between the variables Hilus, PV in Granule Layer and PV in Hilus on GC (all at the rostral level). Model 1 additionally included two theoretical indirect paths: (*i*) MC → PV in Granule Layer → GC and (*ii*) PV in Hilus → MC → GC, with respective indirect and total effects explicitly modeled. Although model 2 (with only direct effects) presents consistently lower WAIC values (differences > 10 in most groups; see Supplementary Table 16), indicating better predictive performance, we chose to focus on the interpretation of model 1, for two main reasons: it is more adherent to the theoretical hypotheses of functional modulation of the dentate gyrus layers by PV+ interneurons; it allows exploring biologically plausible mediations in different experimental conditions. Therefore, we prioritize conceptually informed interpretation, even in the face of predictive penalties. The predictive penalty observed in model 2 may reflect greater structural complexity associated with the introduction of mediation pathways, which require more parameters to be estimated. Furthermore, some experimental groups may exhibit limited variability in indirect effects, which may reduce predictive efficiency even when pathways are theoretically justified (Gelman et al., 2014; Vehtari et al., 2017, Song et al., 2021).

In addition to the comparison between the theoretical models with and without indirect paths, the theoretical model with paths was also evaluated in a multigroup framework, testing two approaches: a restricted version, in which the coefficients were kept equal between the experimental groups, and a free version, in which the parameters were allowed to vary freely between the groups. Both versions were fitted with the blavaan package, using the same data set and a priori specifications. The comparison between the models was performed based on Deviance Information Criterion (DIC). To identify significant effects within each group, we considered standardized coefficients (betas) whose 95% credibility intervals did not include zero (Kruschke, 2014). This approach allowed us to determine which parameters presented robust statistical evidence in each group individually.

In addition, we performed between-group comparisons to investigate whether differences in coefficients between pairs of groups were statistically significant. To do so, we extracted standardized Markov Chain Monte Carlo (MCMC) samples from each group-fit model. We then obtained the set of parameters common to all groups and calculated the distribution of the difference between the corresponding MCMC samples from each pair of groups (Kruschke, 2014). The significance of the differences was assessed based on the 95% credibility interval of the distribution of this difference, considering as significant the one that did not include zero (Kruschke, 2014). The probabilities of the difference being positive or negative were also calculated, being considered relevant when these probabilities exceeded 95% (Gelman et al., 2013). The comparison procedure was performed for all possible pairs among the five groups, allowing the identification of parameters with significant differences between groups.

### 2.5 Behavioral and statistical analysis

Behavioral data were analyzed using the open-source software *DeepLabCut*, through custom scripts developed in Google Colab (Nath et al., 2019). An initial dataset of 200 frames was used to train the network. A *resnet50* network was trained for 100,000 iterations. Then, outlier frames were refined and integrated into the initial frame set. Finally, the data were imported into an open-source package (*Simple Behavioral Analysis*, *SimBA*), from which the total distance traveled, velocity, and the time the animal’s snout was in contact with the region of interest (ROI; objects), as well as entries and exits from the ROI, were extracted (Goodwin et al., 2024). Exploration was defined as occurring when the animal’s snout touched or was < 1 cm from the object. Exploration was not considered when the animal leaned on the object to explore the environment or kept its snout close to the object without exploratory activity.

As a behavioral measure, the cumulative index (*D2 index*) was used. The *D2 index* is calculated from *D1*. The *discrimination index* (*D1*) was calculated as the ratio between the time spent exploring the novel object (*tNO*) minus the familiar object (*tFO*) and the sum of the time spent exploring both objects (*tNO + tFO*). For *D2*, the same formula is applied, but summing the exploration time of each object across trials; thus, for each trial a cumulative index (*D2*) is obtained. Positive *D2* values at the end of the trials represent greater exploration of the novel object. The use of the D2 index is widely adopted in continuous object recognition protocols (Albasser et al., 2010; Ameen-Ali et al., 2012), increases statistical robustness by reducing the impact of point variations in isolated trials and mitigates intra- and inter-individual variability in spontaneous exploration, providing a more stable and representative measure of the animal’s overall performance (Albasser et al., 2010).

Statistical procedures were conducted using SPSS (version 26.0) or R (packages: *dunn.test* and *PMCMRplus*), with differences considered statistically significant at *p* < 0.05. Normality of behavioral and immunofluorescence data was verified using the *Shapiro-Wilk test*. For behavioral parameters, repeated measures and independent *ANOVAs* were used, followed by planned orthogonal contrasts, or the *Kruskal-Wallis test* for independent measures, followed by *Dunn’s post-hoc* test. A contrast analysis was performed to conduct comparisons between each similarity level and conditions involving different objects and all similarity levels (NOR and DIST), as well as comparisons between lower and higher similarity levels. Four contrasts were made as follows: 1. All each level of similarity versus NOR and DIST; 2. 50 and 75% conditions versus 25% condition; 3. 50% experimental condition versus 75%; 4. Conditions: NOR versus DIST. A one-sample *t*-test was used for the discrimination indexes, with a chance value of 0. Effect size (E.S.) was also calculated for each statistical test. For ANOVAs, the effect size used was omega squared (ω^2^): < 0.01 small effect, < 0.06 medium effect, > 0.14 large effect (Cohen, 1988). For direct parametric comparisons, Hedges’ g was used, as it provides a more accurate estimate of effect size in small samples. The classification adopted was: g ≤ 0.2 = small effect, < 0.5 = medium effect, and g ≥ 0.5 = large effect (Hedges and Olkin, 1985). For nonparametric comparisons, Cliff’s Delta (δ) was applied, which is also well-suited for small samples and ordinal data. Its classification follows: δ = 0 = no effect, ± 0.147 = small, ± 0.33 = moderate, and ± 0.474 = large (Cliff, 1993; Meissel and Yao, 2024).

## 3 Results

### 3.1 c-Fos expression occurs differentially in the rostral hippocampus and in layers of the Prh36 in conditions involving novel object recognition and objects with higher similarity

**FIGURE 2:**
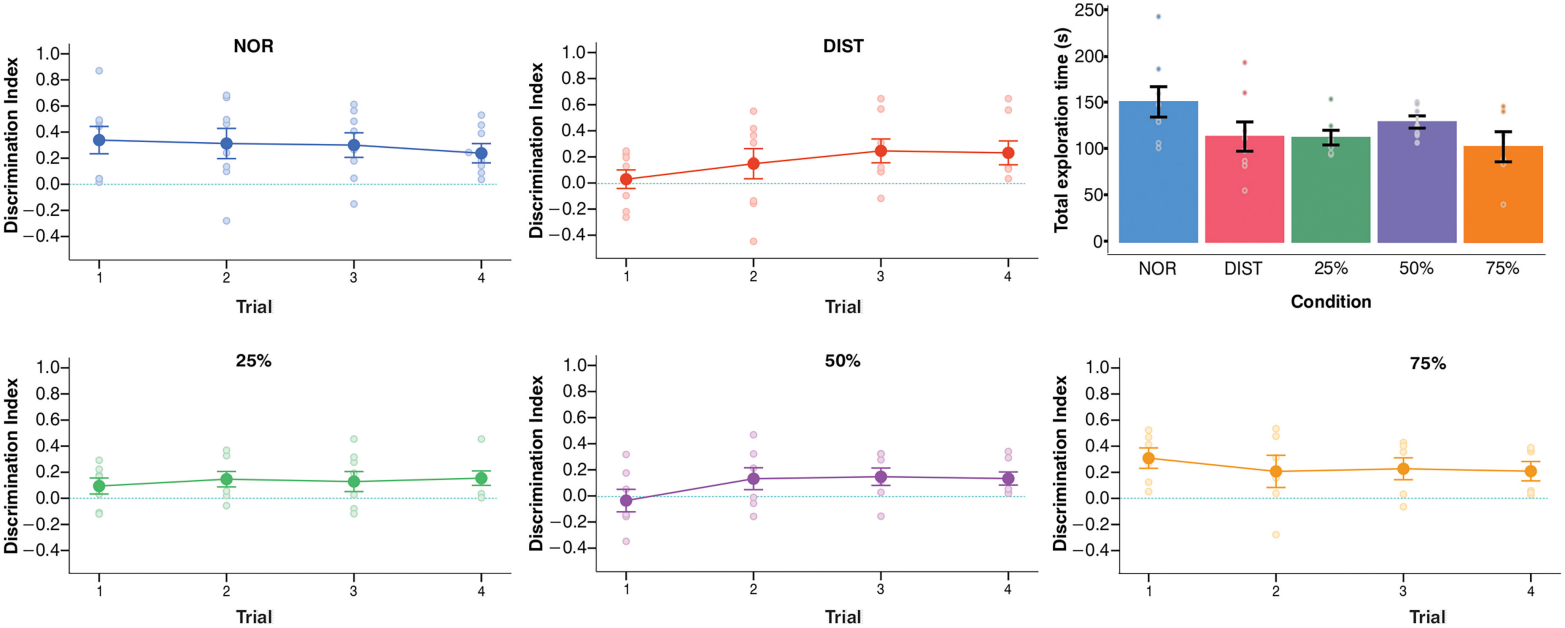
Behavioral results for each experimental condition. Cumulative discrimination index per trial and total exploration time for each experimental condition. The discrimination index is shown in the main panels, and the total exploration time is presented in the upper right corner of each graph. One-sample *t*-tests were applied to evaluate the discrimination index performance against chance. Total exploration times were compared between conditions using ANOVA. Significant differences are indicated by *p* < 0.05; *N* = 6–7 animals per group. Error bars represent the standard error of the mean (SEM).

Evaluating the behavior of animals, in the Novel Object Recognition (NOR) task, animals performed above chance in all sessions, with significantly positive D2 indices: session 1 [*t*(7) = 3.22, *p* = 0.01, *g* = 0.33], session 2 [*t*(7) = 2.69, *p* = 0.03, *g* = 0.36], session 3 [*t*(7) = 3.19, *p* = 0.01, *g* = 0.30], and session 4 [*t*(7) = 3.21, *p* = 0.01, *g* = 0.23]. However, total object exploration time remained stable across sessions [Friedman’s ANOVA: *F*(3, 21) = 0.77, *p* = 0.52], indicating consistent exploratory behavior throughout the trials (Figure 2, top row corresponding to the NOR condition).

In the task involving different levels of object similarity (DIST condition), animals showed significant discrimination only in the last two sessions: session 3 [*t*(7) = 2.80, *p* = 0.02, *g* = 0.29] and session 4 [*t*(7) = 2.63, *p* = 0.03, *g* = 0.29], as detailed in Supplementary Table 1. No significant differences were found in total exploration time across sessions [*F*(3, 21) = 2.30, *p* = 0.16], suggesting that improvements in performance were not driven by increased exploratory activity (see Figure 2), top row corresponding to the DIST condition.

When object similarity was reduced to 25%, animals exhibited significant discrimination performance at the cumulative four sessions, with a D2 index above chance [*t*(6) = 3.73, *p* = 0.014, *g* = 0.08; Figure 2, bottom row corresponding to the 25% similarity condition]. A repeated-measures ANOVA revealed a significant effect of session on total exploration time [*F*(3, 21) = 13.11, *p* = 0.004]. *Post hoc* analyses indicated that session 1 elicited significantly longer exploration compared to session 2 [*t*(7) = 2.14, *p* = 0.01] and session 4 [*t*(7) = 2.14, *p* = 0.01], suggesting progressive familiarization or a shift in exploration dynamics across sessions.

For the 50% similarity condition, animals performed above chance in sessions 3 [*t*(6) = 2.48, *p* = 0.04, *g* = 0.20] and 4 [*t*(6) = 3.01, *p* = 0.02, *g* = 0.15, as shown in Supplementary Table 1]. However, there were no significant differences in total exploration time across sessions [*F*(3, 18) = 1.94, *p* = 0.86], indicating that improved discrimination was not associated with changes in total time of exploration (Figure 1). In the 75% similarity condition, animals displayed above-chance D2 values in sessions 1 [*t*(5) = 3.94, *p* = 0.01, *g* = 0.22], 3 [*t*(5) = 2.71, *p* = 0.04, *g* = 0.24], and 4 [*t*(5) = 2.80, *p* = 0.03, *g* = 0.21]. Nevertheless, object exploration time did not significantly differ across sessions [*F*(3, 21) = 0.25, *p* = 0.88], as shown in Figure 2, bottom row corresponding to the 75% similarity condition.

To account for potential differences in animal performance or motivation, supplementary control analyses were conducted (Supplementary Table 2). A mixed-effects ANOVA revealed no significant main effects of group or session on the discrimination index, nor a significant group × session interaction (see Supplementary Table 2). Similarly, during the sampling phase, the time spent exploring each object (i.e., between-object exploration) did not significantly differ across trials, either within or between experimental groups, and no interaction effects were observed. Specifically, no significant differences were found in total object exploration time between groups [*F*(4, 31) = 1.71, *p* = 0.17; Figure 1A, presented in the upper right panel] or in total distance traveled [*H*(4) = 2.58, *p* = 0.62], indicating that discrimination performance was not confounded by differences in exploratory behavior or locomotor activity.

**FIGURE 3:**
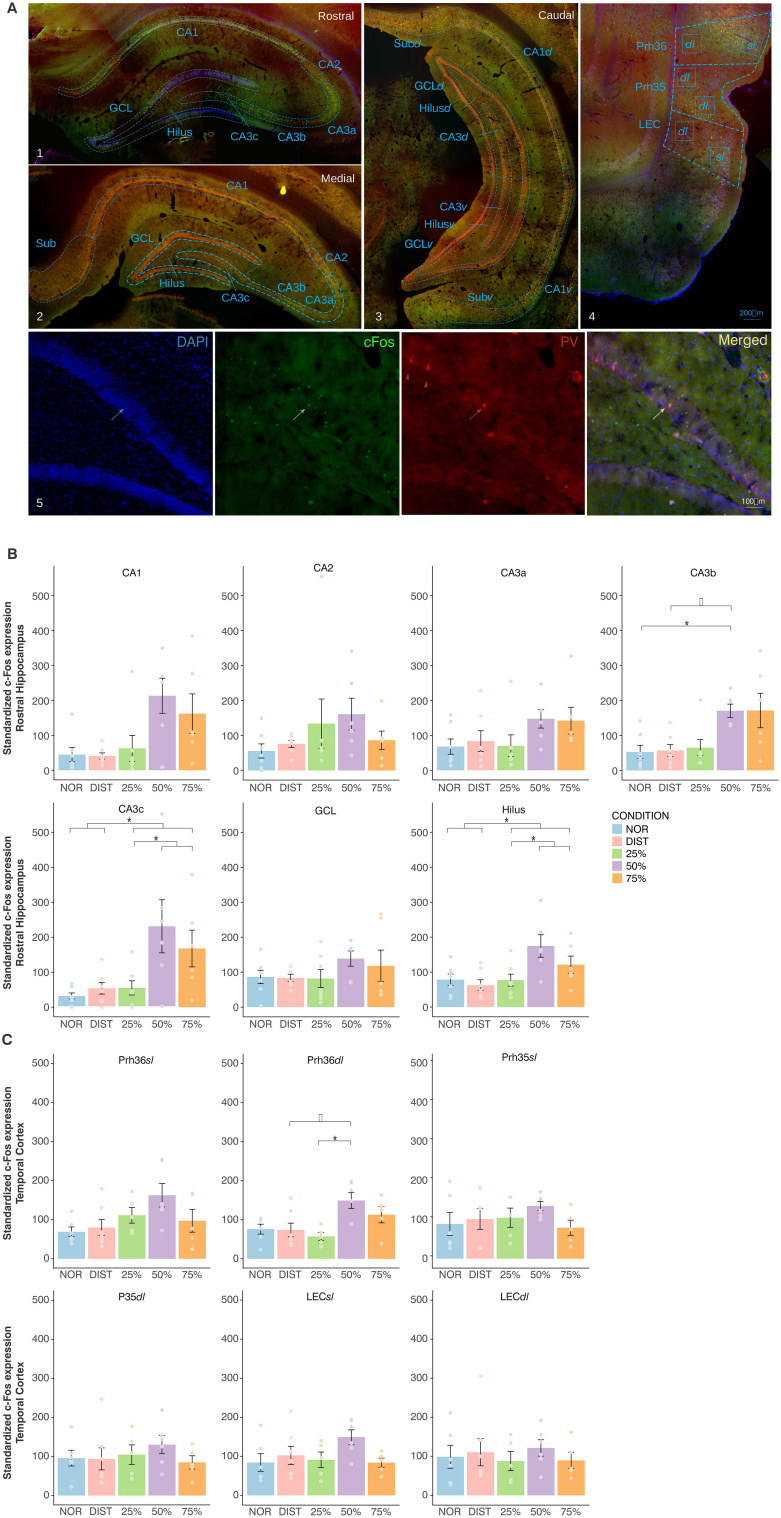
Representative delimitation of hippocampal and parahippocampal regions and c-Fos expression between groups in the rostral hippocampus and parahippocampal cortex. **(A)** Representative delimitation of hippocampal subregions along the rostrocaudal axis (rostral, medial, and caudal—subdivided into dorsocaudal and ventrocaudal) and parahippocampal areas selected for morphometric and neurochemical analyses. Blue lines indicate regional boundaries, and blue squares denote sampling sites in the superficial (sl) and deep (dl) cortical layers. Arrows in the lower panels (from left to right) indicate Pv+/c-Fos+ cells, and red arrowheads indicate Pv+/c-Fos- cells. The rightmost panel displays the merged image of the triple immunostaining. **(B)** Normalized c-Fos expression in subregions of the rostral hippocampus across different similarity conditions. **(C)** Normalized c-Fos expression in subregions and layers of the parahippocampal cortex for the various similarity conditions. Each color corresponds to a specific condition, as indicated in the legend. Each graph corresponds to the subarea of interest. Brackets above the graphs denote the pairs of conditions compared. Depending on data distribution, Kruskal-Wallis tests with Dunn’s *post hoc* comparisons, or planned two-way orthogonal contrasts, were applied. Here only areas with significant differences between groups were represented. *Significant differences are indicated by *p* < 0.05 ‡Denotes trends approaching significance. Sample sizes ranged from *N* = 6–7 animals per group. Error bars represent the standard error of the mean (SEM).

Regarding group comparisons of c-Fos expression in the rostral hippocampus, planned contrasts revealed significant differences in both the CA3c region [*F*(4, 28) = 4.67, *p* = 0.005, ω^2^ = –1.25] and the Hilus layer (GD) [*F*(4, 28) = 3.76, *p* = 0.01, ω^2^ = –0.19] (see Supplementary Tables 3, 4). Specifically, planned contrast tests showed a significant increase in c-Fos expression in CA3c [*t*(28) = 2.84, *p* = 0.008, two-tailed] and Hilus [*t*(28) = 2.87, *p* = 0.001, two-tailed, *g* = –0.91] for the higher similarity conditions (50 and 75%) compared to the 25% similarity condition. Additionally, significant differences were found between the low similarity (25%) and both medium (50%) and high (75%) similarity conditions in CA3cR [*t*(28) = 2.99, *p* = 0.006, two-tailed, *g* = 0.95] and Hilus [*t*(28) = 2.75, *p* = 0.01, two-tailed, *g* = –1.08]. No significant differences were detected between the 50 and 75% conditions, nor between the NOR and DIST groups (see Figure 3B and Supplementary Tables 3, 4).

A significant effect was also observed in c-Fos expression within the CA3b subregion [*H*(4) = 12.49, *p* = 0.01, ω^2^ = 0.18]. *Post hoc* Dunn’s tests revealed a significant increase with a large effect size in CA3b expression when comparing the NOR and 50% similarity conditions (*p* = 0.04, *z* = –0.87, δ = –0.85). A trend toward increased c-Fos expression in CA3b was also noted between the DIST and 50% similarity conditions (*p* = 0.05, *z* and δ = –0.85) (see Figure 3). No other rostral hippocampal subregions showed statistically significant differences. Furthermore, when analyzed separately, no significant differences in c-Fos expression between experimental groups were observed within the medial, caudal-dorsal, or caudal-ventral subregions of the hippocampus (see Supplementary Tables 3, 4). Importantly, we did not perform direct comparisons across hippocampal subregions (e.g., dorsal vs. ventral); instead, each subregion was analyzed independently across experimental groups.

Together, these results suggest that c-Fos expression in CA3c and Hilus of the rostral hippocampus is modulated by the level of object similarity, increasing as the task demands greater feature disambiguation compared to conditions with lower similarity. In contrast, c-Fos expression in CA3b appears selectively involved in the discrimination process under medium similarity demands. Notably, these expression patterns were confined to the rostral hippocampus.

Regarding the perirhinal cortex (Prh36*dl*), Kruskal-Wallis tests identified a significant difference among conditions [*H*(4) = 10.25, *p* = 0.04, ω^2^ = 0.21]. Dunn’s *post hoc* comparisons indicated that the 50% similarity condition exhibited higher c-Fos expression relative to the 25% condition (*p* = 0.03, *z* = –2.10, δ = –0.86). There was also a trend toward increased expression in the 50% condition compared to DIST (*p* = 0.06, *z* = –2.68, δ = –0.76) (see Supplementary Table 3 and upper line of Figure 3C). These findings imply that recognition of similar objects elicits modulation of c-Fos expression in the perirhinal cortex.

Correlations between discrimination indices and c-Fos expression were conducted only for areas that showed differences between groups. We found significant negative correlations for the NOR condition, between the discrimination index and c-Fos expression (*r* = –0.88). Strong significant correlations were also found between D2 and c-Fos expression in CA3b (*r* = 0.94) and CA3c (ρ = 0.93) (see Supplementary Figure 2). These findings highlight the specific involvement of CA3 in the behavioral task and may reflect its role in pattern separation and completion processes. The absence of a significant correlation in the hilus suggests that, although this region is involved in general hippocampal processing, its c-Fos activation does not directly track discrimination performance under the conditions tested.

### 3.2 The parahippocampal-rostrocaudal hippocampus network interacts differently and more integratively depending on the similarity level of the recognition task

**FIGURE 4:**
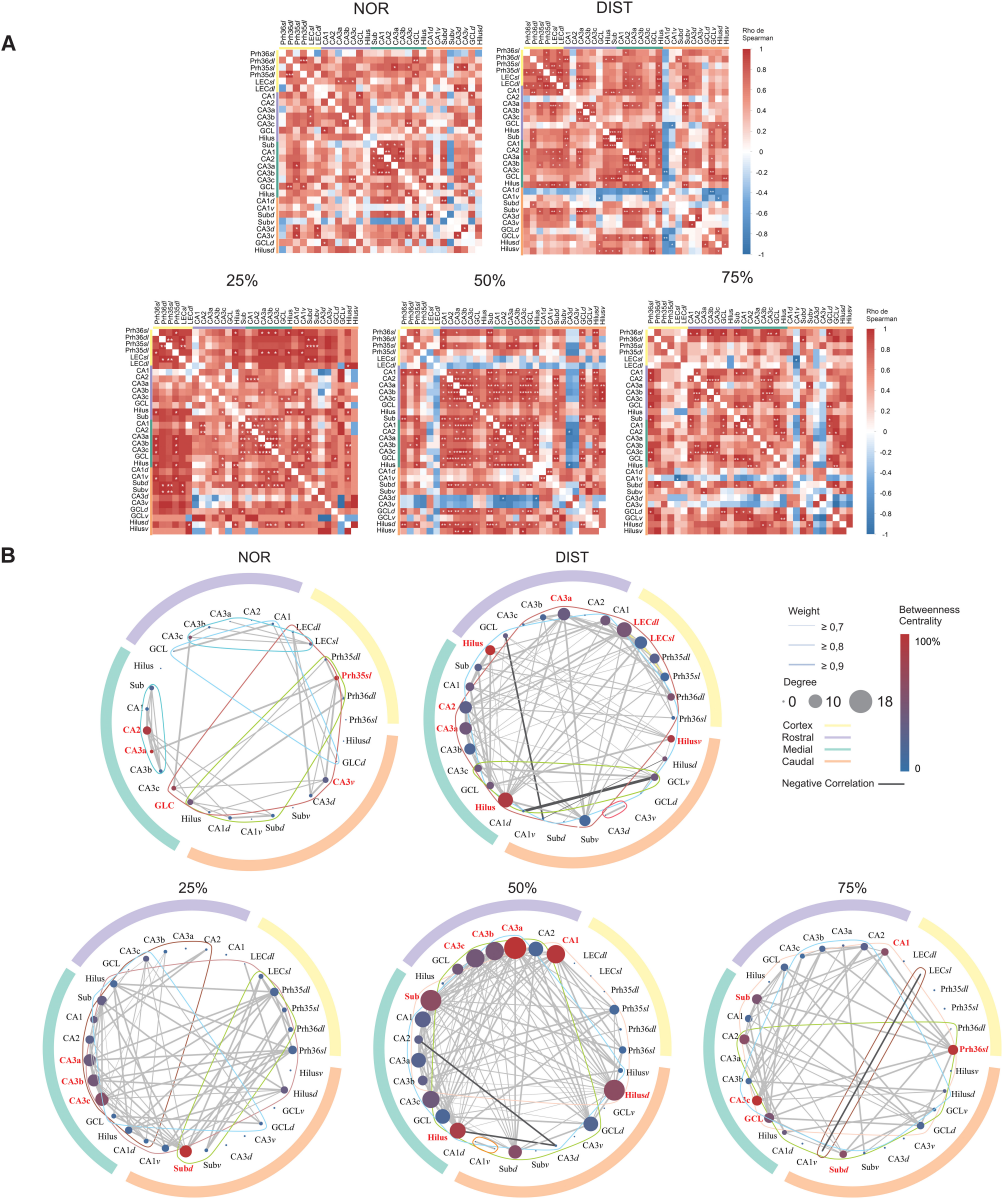
Network analysis of all c-Fos+ activated cells in the hippocampal and parahippocampal regions under different similarity conditions. **(A)** Correlation matrix constructed from Spearman correlations between medial temporal lobe areas at different rostrocaudal-dorsoventral hippocampus levels for each experimental group. Warm colors indicate stronger positive correlations, cold colors indicate stronger negative correlations. *Significant correlations at *p* < 0.05. **Significant correlations at *p* < 0.01. ***Significant correlations at *p* < 0.001. (**B**) Graphs constructed for each group. Outer circle with different colors representing different portions of the rostrocaudal axis of the hippocampus, see matches in the upper right. Inside the graph, each circle corresponds to a node (region). Edges represent correlations between areas. Lines around a graph represent the number of clusters in the graph. In the upper right is explained, Edge thickness represents the strength of the correlation. Darker lines represent negative correlations. Colors represent different levels of the rostrocaudal axis along the hippocampus. Node colors represent centrality levels among them. Node size represents degree. Hubs in each network have their names highlighted in red. *N* = 6–7.

In addition to identifying differences in c-Fos expression between brain areas, we sought to obtain a more holistic view of the functional interactions between these regions under different levels of interference. To investigate how the parahippocampal regions and the distinct anteroposterior levels of the hippocampus are organized into a network in each experimental condition, we performed a graph-based analysis. The graphs constructed from the c-Fos expression correlations revealed 34 significant connections in the NOR condition and 91 connections in the DIST condition. For the intermediate conditions of 25, 50, and 75%, 69, 123, and 54 connections were observed, respectively (see Figure 4A).

To compare the level of coordinated activity between conditions, considering the correlation matrices, a Chi-square test was conducted between groups with subsequent Chi-square tests corrected for the number of correlations between groups. The Chi-square test showed a significant difference between groups [X^2^(4) = 65.43, *p* < 0.001]. The corrected pairwise Chi-square tests found differences between all conditions, with the 50% and DIST conditions having the highest proportions of correlations, 0.26 and 0.19, respectively. It should be noted that the DIST, 50, and 75% conditions showed negative correlations.

To measure the relevance of individual nodes within the networks and to obtain information on how information flows within them, degree centrality, betweenness centrality, and strength were calculated (see Supplementary Tables 5, 6). Nodes were ranked according to each metric to reveal their relative importance in the respective network. In the NOR condition, nodes from CA2 (highest degree and strength), CA3a, CA3c, GCL, belonging to the medial hippocampus, Prh35*sl* (highest centrality), and caudal CA3*v* stood out as hubs. It is interesting to note that much of the medial hippocampus was central in this condition, with the Prh35*sl* region being an important bridge between clusters; see first circle graph (NOR) in Figure 4B. Medial region highlighted by green outer circle and high interconnection between its regions (see Supplementary Table 5).

For the DIST condition, the regions considered hubs were the rostral Hilus (highest centrality), medial Hilus (highest degree), ventral Hilus, and LEC*dl* (highest degree and strength). It is notable that the Hilus in different anteroposterior portions of the hippocampus was engaged in this task as important hubs of the rostral hippocampus and bridges between clusters of the rostral-medial and caudal hippocampus, see hub areas highlighted in red in the second circle graph in Figure 4B.

At 25% similarity, all portions of the medial CA3 were hubs: CA3a, CA3b, CA3c (highest degree), and the caudal Subiculum*dl* (highest centrality and strength). The medial CA3 subportions connect different portions of the hippocampus and parahippocampal region. The caudal Subiculum*dl*, in turn, seems to be a central bridging region connecting the parahippocampal region with the medial and rostral hippocampus, see hub areas highlighted in red and cluster highlighted by colored connected lines in green, brown and dark red the third circle graph of Figure 4B.

When evaluating 50% similarity conditions, some rostral hippocampal substructures are considered hubs: CA1, CA3a (highest degree, centrality, and strength), CA3b, and CA3c. The Subiculum and Hilus nodes of the medial hippocampus, as well as the caudal Hilus*d*, are also considered hubs. It is interesting to note that the caudal Hilus*d* and medial Subiculum seem to connect a large cluster involving mainly the medial hippocampus and parahippocampal region. The rostral CA3 subareas, together with the medial Hilus, are central for connecting another cluster that includes rostral, caudal, and ventral hippocampal regions, see hub areas highlighted in red and cluster highlighted by colored connected lines in green, blue and orange within the fourth circle graph of Figure 4B.

In the highest similarity condition (75%), some medial hippocampal subregions stand out as hubs: Subiculum, CA3c (highest centrality), and GCL. Besides these, the caudal Subiculum*dl*, rostral CA1, and Prh36*sl* (highest strength) were also considered hubs. Except for CA1 and Subiculum*c*, all hubs had the same degree (8). The medial CA3c is an important hub for connecting rostral hippocampal areas with nodes from other hippocampal portions. Prh36*sl*, in turn, is the only cortical area strongly engaged in the network, serving as a hub of strong connections from different hippocampal portions to the parahippocampal region, working together with other hubs in these regions such as rostral CA1 and medial GCL; see the lower right corner of Figure 4B; hubs highlighted in red.

The global efficiency analysis showed a difference between the DIST condition and all other conditions (*p* < 0.01). The other conditions showed no differences between them. The standardized efficiency for each condition was: NOR = 0.71; DIST = 0.92; 25% = 0.72; 50% = 0.74; and 75% = 0.62.

When comparing the global clustering coefficients, we observed differences between the NOR condition and all other conditions (*p* < 0.0001). There were also differences between the 25 and 50% conditions (*p* < 0.0001) and between 50 and 75% (*p* < 0.001). The global clustering coefficient for each condition was: NOR = 7.89; DIST = 2.79; 25% = 3.42; 50% = 2.24; and 75% = 3.50. Network clustering is highest in the NOR condition, indicating stronger local connectivity, with variations among other conditions reflecting differences in network organization (see Supplementary Table 7).

Using the Louvain clustering algorithm, variations in cluster structure were observed under different experimental conditions. Excluding areas without connections, each condition consistently presented 4 clusters, except the NOR condition which had 5 clusters (see Supplementary Table 8 and Figure 4, colored lines within the graphs). Although the number of clusters was quite similar between networks, modularity value can be used as a metric of cluster homogeneity in each network. The modularity value differed between the 50% condition and all conditions (*p* < 0.01), with no difference between the other conditions. The modularity value suggests that the 50% network is more homogeneous, with no clear divisions within the community, which may indicate a highly distributed network without substructures. Furthermore, the conditions with similar new objects showed lower modularity than the new object condition. A lower modularity value corresponds to greater homogeneity in the network (see Supplementary Table 9 and Supplementary Figure 3).

### 3.3 The expression of activated interneurons (PV+/c-Fos+) is increased in higher similarity conditions along the rostrocaudal axis of the hippocampus

**FIGURE 5:**
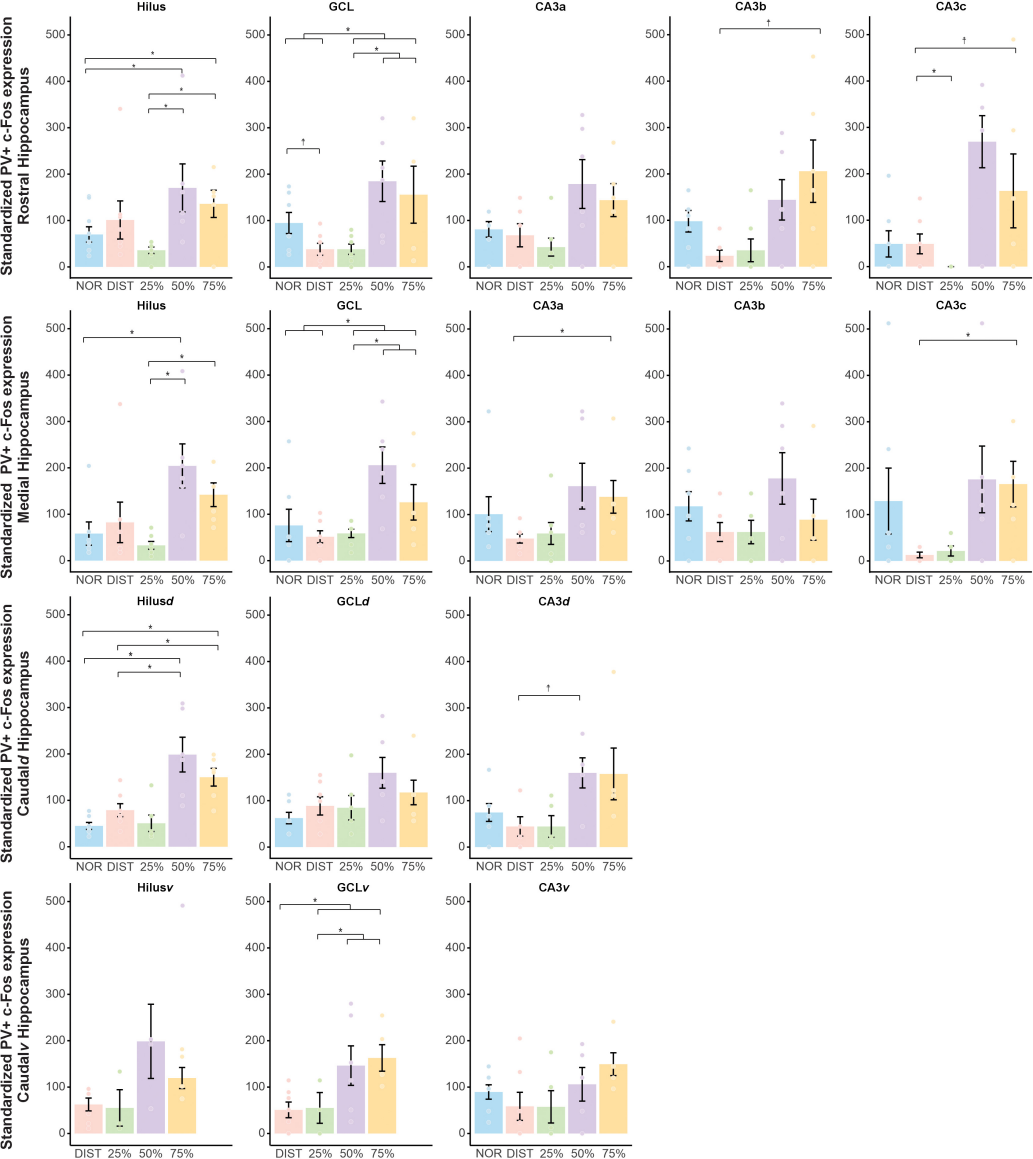
Interneuron recruitment (PV+/c-Fos+) between groups in the DG-CA3 microcircuit along the dorsoventral anteroposterior axis of the hippocampus. Normalized c-Fos expression in DG layers and different CA3 portions in the rostral, medial, caudal dorsal, and caudal medial hippocampus across experimental conditions. Each line of graphs in the figure represents a portion of the hippocampus. Each graph corresponds to a subarea of the hippocampus. Each color corresponds to a specific condition, as indicated in the legend. Brackets above the graphs denote the pairs of conditions compared. Depending on data distribution, Kruskal-Wallis tests with Dunn’s *post hoc* comparisons, or planned two-way orthogonal contrasts, were applied. *Significant comparisons considering *p* < 0.05. ‡ Trends approaching significance; *N* = 6–7. ± Standard error of the mean.

Inhibitory activity has been shown to be central to pattern separation processes, so we sought to see whether PV activity changed in the DG-CA3 microcircuit across conditions. When analyzing PV+/c-Fos+ expression in the subregions of the rostral hippocampus (Supplementary Table 10 and Figure 5, first line), a statistically significant difference was found in the Hilus [*H*(4) = 12.48, *p* = 0.014, ω^2^ = 0.55]. There was greater expression in the 50% (*z* = –3.31, *p* = 0.005, δ = –0.57) and 75% (*z* = –3.05, *p* = 0.009, δ = –0.10) conditions compared to the NOR condition. The 50% (*z* = –3.19, *p* = 0.006, δ = 0.02) and 75% (*z* = –3.05, *p* = 0.009, δ = 0.57) conditions also showed higher expression than the 25% condition.

In the GCL rostral, ANOVA indicated differences between groups [*F*(4, 28) = 3.95; *p* = 0.01, ω^2^ = –1.33]. The experimental conditions of 25, 50, and 75% exhibited higher PV+/c-Fos+ expression than the NOR and DIST conditions [*t*(28) = 2.46, *p* = 0.02 (bilateral), *g = –*0.62]. There was also an increase in PV+/c-Fos+ expression in the higher similarity conditions (50 and 75%) compared to the lower similarity (25%) [*t*(28) = 3.33, *p* = 0.007 (bilateral), *g* = 1.18]. A trend toward difference was observed between the conditions NOR and DIST [*t*(28) = –2.16, *p* = 0.057 (bilateral), *g* = 1.08], with DIST having higher PV+/c-Fos+ expression (see Figure 5 and Supplementary Table 11).

In the CA3b region, differences between groups were observed [*H*(4) = 11.98, *p* = 0.017, ω^2^ = 0.23], where the 75% condition had greater PV+/c-Fos+ expression than the DIST condition (*z* = –2.68, *p* = 0.03, δ = –0.04). For the CA3c region, group differences were found [*H*(4) = 13.37, *p* = 0.01, ω^2^ = 0.31], specifically with DIST showing more PV+/c-Fos+ expression than 25% (*z* = –2.68, *p* = 0.03, δ = –0.76). A trend was observed between 25 and 75% (*z* = –2.51, *p* = 0.05, δ = 0.73), with 75% exhibiting greater PV+/c-Fos+ expression. No differences were observed in CA3a, to view the raw PV data (see Supplementary Tables 18–20).

For the medial hippocampus portion (Figure 5, second line), differences between groups were found in the Hilus [*H*(4) = 15.34, *p* = 0.004, ω^2^ = 0.38]. This difference was observed between the 25 and 75% conditions (*z* = –2.80, *p* = 0.02, δ = 0.66), with higher PV+/c-Fos+ expression in the 75% condition. There was also higher PV+/c-Fos+ expression in the 50% condition compared to 25% (*z* = –3.11, *p* = 0.009). The 50% condition also had greater PV+/c-Fos+ expression than the NOR condition (*z* = –2.50, *p* = 0.049, δ = –0.80). For the GCL, a significant increase in PV+/c-Fos+ was found between groups [*F*(4, 28) = 4.77; *p* = 0.005, ω = –1.23]. PV+/c-Fos+ expression was significantly higher in the similarity conditions (25, 50, and 75%) compared to NOR and DIST [*t*(28) = 2.99, *p* = 0.007 (bilateral), *g = –*0.82]. A significant increase was also found between the higher similarity conditions (50 and 75%) compared to the lower similarity (25%) [*t*(28) = 3.70, *p* = 0.003 (bilateral), *g* = 1.31, see Supplementary Table 11]. Differences were observed in the CA3a area [*H*(4) = 12.06, *p* = 0.01, ω ^2^ = 0.26], where the 75% group had higher PV+/c-Fos+ expression than the DIST group (*z* = 2.64, *p* = 0.04, δ = 0.80). For CA3c, a difference was also found [*H*(4) = 11.98, *p* = 0.01, ω^2^ = 0.25] with increased PV+/c-Fos+ in the 75% group compared to DIST (*z* = 2.58, *p* = 0.04). No differences were seen in CA3b (see Figure 5).

Regarding the caudal-dorsal hippocampus portion (Figure 5, third line), differences in PV+/c-Fos+ expression were found in the Hilus*d* [*H*(4) = 20.07, *p* = 0.001, ω^2^ = 0.25]. There was an increase in PV+/c-Fos+ expression in the 50% condition compared to NOR (*z* = –3.55, *p* = 0.036, δ = –0.97), as well as an increase in the 75% condition compared to NOR (*z* = –2.90, *p* = 0.01, δ = 0.57). A significant increase in PV+/c-Fos+ expression was observed in the 50% condition compared to DIST (*z* = 3.36, *p* = 0.003, δ = –0.89), as well as an increase in the 75% condition compared to DIST (*z* = –2.93, *p* = 0.01, δ = –0.54). For CA3*d*, a significant difference was found using Kruskal-Wallis [*H*(4) = 10.65, *p* = 0.03, ω^2^ = 0.20], but only a trend was seen with increased expression in the 50% condition compared to DIST (*z* = 2.56, *p* = 0.05, δ = –0.76). No differences were found in GCL*d*.

For the caudal-ventral hippocampus subareas (Figure 5, last line), a significant difference was observed in the GCL*v* [*F*(4, 28) = 3.49; *p* = 0.03, ω^2^ = –1.29], but only trends were found between NOR and all similarity conditions (25, 50, and 75%) [*t*(28) = 2.07, *p* = 0.05 (bilateral), *g* = –0.92]. There was also a trend between the lower similarity (25%) and higher similarity conditions (50 and 75%) [*t*(28) = 2.1, *p* = 0.05 (bilateral), *g* = 1.22], with the higher similarity conditions showing greater expression (see Figure 5).

### 3.4 The c-Fos+ activated cells versus PV+/c-Fos+ activated interneuron network in the DG-CA3 circuit across different hippocampal segments is modulated by similarity conditions

**FIGURE 6:**
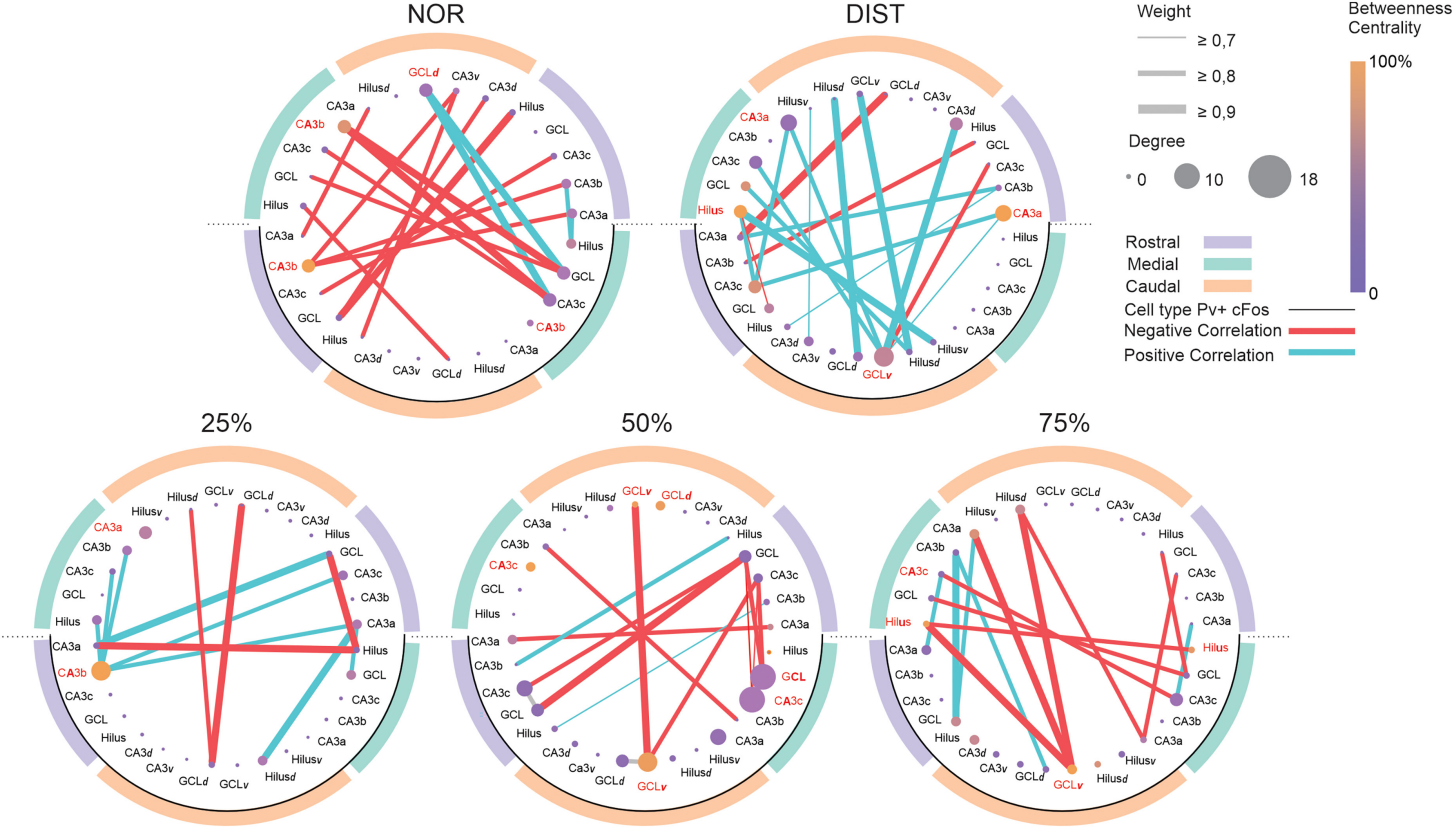
Network analysis between all c-Fos+ activated cells versus PV+/c-Fos+ inhibitory activated cells in the DG-CA3 microcircuit across different anteroposterior and dorsoventral portions of the hippocampus under varying similarity conditions. Graphs constructed for each group. The outer circle with different colors represents different anteroposterior portions of the hippocampus. The lower half of each graph, outlined with a black line, represents PV+ interneurons; the upper half represents excitatory neurons. Within the graph, each circle corresponds to a node (region). Edges represent correlations between areas. The thickness of the edges represents the strength of the correlation. Red lines represent negative correlations; blue lines represent positive correlations. Colors denote different anteroposterior hippocampal portions. Node colors represent their centrality level. Node size represents degree. Network hubs have their names highlighted in red. *N* = 6–7.

Beyond just looking at interactions in general, we wanted to see how interactions between PV+ interneurons and other cell types occurred at different levels of similarity. When observing the network generated from the correlation data between inhibitory and excitatory cells, it was noted that the direction of activity between areas changes in terms of the proportions of positive and negative correlations [*X*^2^(4) = 37.717, *p* < 0.001], with notable and significant differences in the proportion of negative and positive correlations between the NOR and DIST conditions [*X*^2^ = 9.11, *p* = 0.02], DIST and 50% [*X*^2^ = 10.45, *p* = 0.002], and DIST and 75% [*X*^2^ = 5.72, *p* = 0.01], with the DIST condition being more active than all the respective conditions. Differences were also observed between the 50% and NOR conditions (NOR being less active) (*p* < 0.05), and between the 25 and 75% conditions (25% being less active) (*p* < 0.05) (see Figure 6).

In the condition NOR, a predominance of negative correlations between c-Fos+ and PV+/c-Fos+ cells was observed, suggesting robust inhibitory control exerted by PV interneurons. In contrast, the DIST condition showed a marked increase in positive correlations between c-Fos+ and PV+/c-Fos+ cells, accompanied by a reduction in negative ones, indicating a possible release from inhibitory control. In the 25–75% conditions, there was dynamic reorganization of connections in each condition. Specifically, in the 50% condition, most correlations between those cells were negative and directed toward the DG-CA3 microcircuit of the rostral hippocampus, while 25% (fewest correlations) and 75% showed correlations directed toward the medial hippocampus, reflecting functional configurations specific to each experimental context (see Figure 6). Notably, the 50% condition had the highest proportion of negative correlations (0.83), while the DIST condition had the highest proportion of positive correlations (0.83) (for centrality metric and hubs see Supplementary Tables 12, 13).

Additionally, there were differences in global efficiency across all conditions (*p* < 0.01). Differences were also found in the clustering coefficient across all conditions (*p* < 0.01), with the 50% condition showing the highest global efficiency (0.35) and clustering coefficient (0.10) (see Supplementary Table 14). Modularity analysis revealed significant differences across all conditions (*p* < 0.01), with the 50% network having the lowest normalized modularity value (0.40) (see Supplementary Table 15). Taken together, these findings may suggest that the excitatory-inhibitory balance in the DG-CA3 circuit is modulated. Inhibition mediated by PV interneurons is not static but undergoes dynamic and condition-specific adjustments, reflecting the circuit’s functional plasticity and adaptive responses to different demands.

### 3.5 Activated cells in the GCL and Hilus may exert inhibitory/excitatory effects on GCs depending on novel object similarity

To understand how different cell types within the dentate gyrus–CA3 microcircuit interact to resolve varying levels of stimulus similarity, we tested a theoretical model assessing these relationships across experimental groups. Specifically, we aimed to identify the functional modulation patterns involving activated cells in the granule layer (GCs), activated cells (possible mossy cells—MCs) in the Hilus, and Parvalbumin-positive (PV+) interneurons in distinct layers, and how these contribute to pattern separation performance.

To assess the stability of these effects across groups, the theoretical model was tested in two versions: a restricted model with structural parameters constrained to equality across groups, and a free model allowing parameters to vary between groups. Model comparison using the Deviance Information Criterion (DIC) indicated superior fit for the free model (DIC = 170.93) over the restricted model (DIC = 182.54). This finding supports the presence of significant differences in latent variable relationships across groups. The selected free model demonstrated satisfactory fit for all groups, with all parameters converging adequately ($\hat{\text{R}}$ < 1.01). Therefore, all subsequent analyses employed this model (adjusted fit indices per group are provided in Supplementary Table 16).

The proposed theoretical model examined how activity within the granule cell layer in the DG is modulated both directly and indirectly by influences from activated cells in the Hilus and PV+ interneurons in various layers. Analyses conducted separately for each experimental group revealed distinct patterns related to behavioral performance in pattern separation tasks.

In the NOR group, the sole significant path was a direct negative effect of PV interneurons in the Hilus on mossy cells (β = –0.70, 95% CI: –0.95 to –0.25), indicating that higher PV expression is associated with reduced residual MC activity, consistent with effective local inhibition. No significant direct or mediated effects on granule cells were observed, suggesting that without stimulus overlap, this microcircuit does not exhibit meaningful functional modulation downstream. This absence of effects may reflect that local inhibition in the Hilus, while present, does not translate into substantial changes in granule cell activity under these task conditions.

In the DIST group, two significant effects emerged. A direct inhibitory effect of PVs in the Hilus on MCs was again present (β = –0.15, 95% CI: –0.36 to –0.009), though with smaller magnitude than in NOR. Additionally, a significant positive effect from MCs to PV interneurons in the granular layer was observed (β = 0.52, 95% CI: 0.07–0.83). No other direct or mediated effects reached statistical robustness, indicating a pattern resembling NOR, with local inhibition in the Hilus but no detectable downstream consequences in granule cells.

**FIGURE 7:**
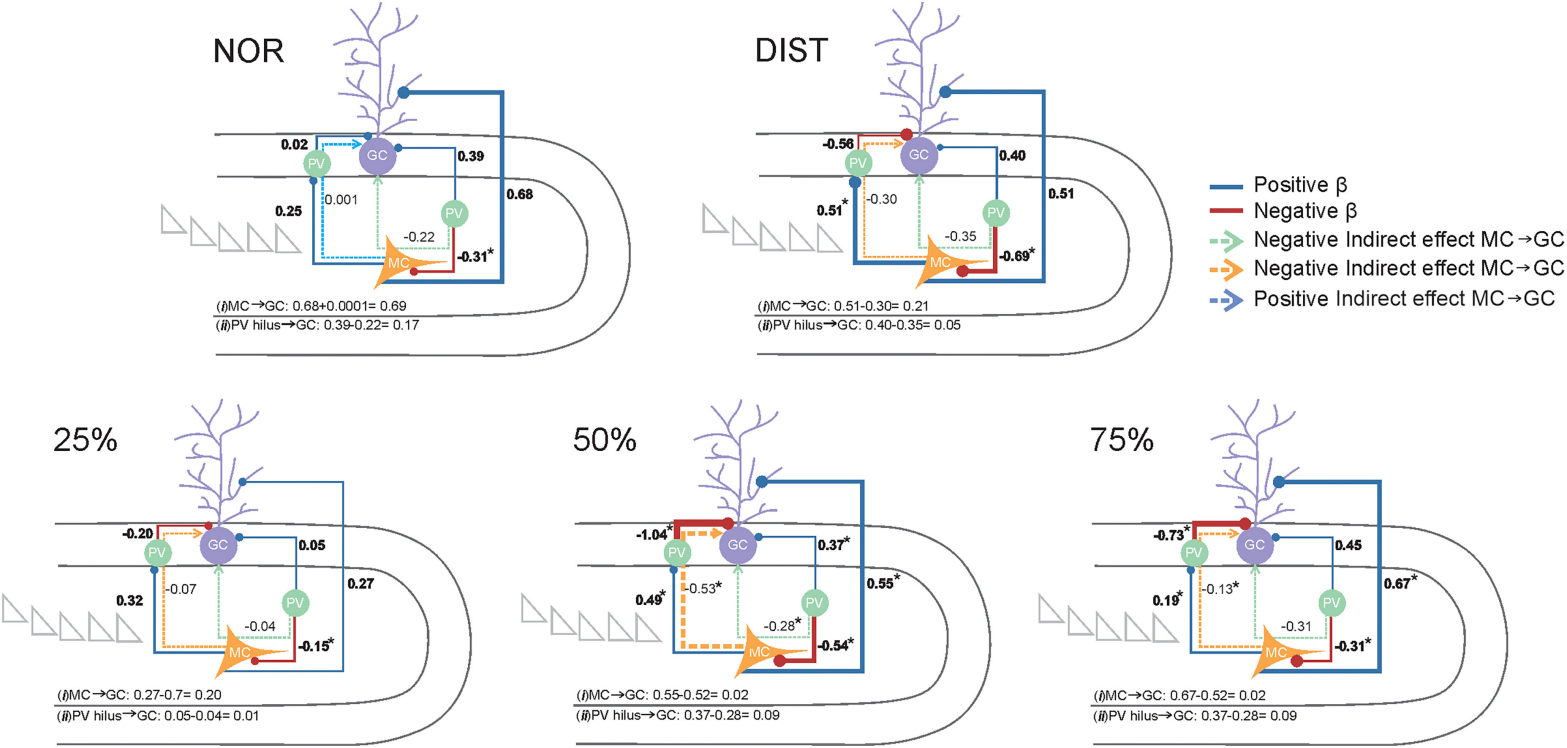
Representation of the Bayesian structural equation model depicting theoretical relationships regarding the modulation of all c-Fos+ activated cells and PV+/c-Fos+ activated interneurons located in the DG layers on the activity of cells in the granule layer. Each experimental condition is represented by a distinct structural equation model (conditions identified above each model). Activated cells - possible Mossy cells (MCs) - located in the Hilus are highlighted in yellow. Parvalbumin-positive (PV+) interneurons in both the Hilus and granule cell layer are highlighted in green, while activated cells in the granular layer (GCs) are shown in lilac. Solid lines represent modeled pathways, with circles at the end of each path indicating the target variable of the effect. Positive β coefficients are depicted in blue, and negative β coefficients in red. Circles at the end of each path represent the target location of the connection. Indirect pathways are illustrated by dashed arrows: indirect pathways from PV interneurons in the Hilus to GCs are shown in green, whereas indirect positive or negative pathways from MCs to GCs are shown in blue and yellow, respectively. Each modeled path is accompanied by its corresponding standardized β value. The β value of Direct effects betas are highlighted in bold, other β value are indirect effects. Total effect paths are shown at the bottom of each model. Total effect paths were computed as follows: (i) MC → GC (direct effect plus or minus indirect effects) and (ii) PV in Hilus → GC (direct effect plus or minus indirect effects), with the sign of the total effect determined by the direction of the indirect effect. Asterisks (*) indicate significant β values, defined as those whose credible intervals do not cross zero. Total effect values for MCs on GCs and PV interneurons in the Hilus on GCs are shown at the bottom of each model. Sample sizes ranged from *N* = 6–7.

Similarly, the 25% similarity group showed only the direct inhibitory effect of Hilus PVs on MCs (β = –0.70, 95% CI: –0.95 to –0.25), with no significant modulation of granule cells. This reinforces that under low similarity demands, the microcircuit’s functional modulation remains limited, dominated by local inhibitory interactions within the Hilus (see Figure 7 and Supplementary Table 17).

In contrast, the 50% similarity group exhibited a more complex and integrated functional profile, characterized by multiple significant effects (Figure 7). These included a positive direct effect of mossy cells (MCs) on granule cells (GCs) (β = 0.55, 95% CI: 0.16–1.10); a negative direct effect of PV interneurons in the granule cell layer on GCs (β = –1.05, 95% CI: –1.33 to –0.83); a positive direct effect of PV interneurons in the Hilus on GCs (β = 0.37, 95% CI: 0.07–0.67); a negative indirect effect of MCs on GCs mediated by PV interneurons in the granule cell layer (β = –0.53, 95% CI: –1.07 to –0.13); and one significant negative indirect effect of PV interneurons in the Hilus on GCs via MCs (β = –0.29, 95% CI: –0.61 to –0.05).

This pattern of effects suggests that, under moderate stimulus similarity, the dentate gyrus microcircuit achieves a dynamic balance between excitation driven by MCs and inhibition mediated by PV interneurons in both the Hilus and granule cell layer. The negative mediation through PV interneurons indicates a refined inhibitory control over granule cell activity, reflecting a finely tuned microcircuit engagement under these conditions (see Figure 7).

Similarly, the 75% similarity group also displayed a functionally engaged pattern, albeit with somewhat lower magnitude effects. Significant paths included a positive direct effect of MCs on GCs (β = 0.67, 95% CI: 0.06–1.13); a negative direct effect of PV interneurons in the granule cell layer on GCs (β = –0.74, 95% CI: –0.96 to –0.21); a negative direct effect of PV interneurons in the Hilus on MCs (β = –0.47, 95% CI: –0.78 to –0.08); and a negative indirect effect of MCs on GCs mediated by PV interneurons in the granule cell layer (β = –0.14, 95% CI: –0.33 to –0.007). Despite the lower magnitude compared to the 50% group, these results similarly reflect a balance between direct excitatory inputs and inhibitory modulation, particularly through the MC → PV (granule layer) → GC pathway.

The proportion of explained variance (R^2^) in granule cell activity varied among groups, with the highest values observed in the 50% (96.3%) and 75% (89.3%) similarity groups. Lower explained variances were seen in the NOR (80.7%), 25% (75.1%), and DIST (64.8%) groups. These differences suggest that the model captures granule cell activity more accurately under conditions of intermediate and high stimulus overlap, possibly reflecting increased engagement of the modeled regulatory microcircuitry.

Although cross-group comparisons of direct and indirect regression coefficients (β values) did not reveal statistically significant differences (all confidence intervals included zero), distinct within-group patterns emerged. Notably, the 50 and 75% similarity groups were the only ones in which all modeled pathways reached statistical significance. This pattern was absent in groups exposed to distinct objects (NOR), mixed similarity levels (DIST), or low overlap (25%). These findings imply that, despite the lack of formal between-group differences, the functional mechanisms—such as PV interneuron influence and hilar modulation of granule cells—become consistently engaged under intermediate similarity demands, orchestrating a balanced excitatory-inhibitory control over granule cell activity.

## 4 Discussion

Taking advantage of rodents’ natural tendency to explore novelty, several paradigms have been developed to assess memory in animals, primarily considering their significant preference for novel objects compared to familiar ones (Ennaceur and Delacour, 1988; Cohen and Stackman, 2015; Blaser and Heyser, 2015). However, beyond simply recognizing, unique representations of similar events that can be associated with different outcomes are also necessary, through a process known as pattern separation (Josey and Brigman, 2015; Gilbert and Kesner, 2006; Miranda et al., 2017). Therefore, the aim here was to assess the brain circuits underlying the recognition of objects with different degrees of similarity, using a multi-trial protocol.

To this end, c-Fos expression was evaluated between groups at different rostrocaudal levels of the hippocampus (Rostral, Medial, Caudal-dorsal, and Caudal-ventral), as well as in distinct layers of the parahippocampal region. Considering only animals that successfully discriminated in all conditions, c-Fos expression in the rostral portion of the hippocampus—specifically in the rostral Hilus and in the CA3 subregions (CA3b and CA3c)—was found to be dependent on the level of similarity, increasing as the task required greater disambiguation of features compared to tasks with lower levels of similarity. These results support hypotheses that position the dentate gyrus and CA3 as central nodes in a refined network that operates in the disambiguation of similar features, specifically for objects (Burke et al., 2018; Senzai, 2018; Stark and Stark, 2017). We infer that the observed effects on c-Fos and PV are not solely due to exposure to the environment, as the presence of the object was the only explicit difference between groups, and previous studies using object recognition tasks have shown that neural activation cannot be explained by context alone (Barbosa et al., 2013). However, it is important to note that we cannot currently determine whether the observed modulation in connectivity is specific to the NOR task or could also occur in other novelty-based tasks, such as those involving a novel context, representing a limitation of the present study and an avenue for future research to explore.

Interestingly, the Hilus appears to be sensitive to changes at all levels of similarity, which aligns with findings that suggest this layer of the gyrus shows increased activation in response to environmental changes, making this region crucial for pattern separation (Senzai and Buzsáki, 2017; GoodSmith et al., 2022). These findings are consistent with those reported by Bernstein et al. (2019), where differences were observed in the Hilus but not in the granule cell layer under varying novelty conditions. These data implicate the role of mossy cells in the circuits responsible for distinguishing between new and similar objects, highlighting their importance in disambiguating overlapping memories (Huang et al., 2024).

Another noteworthy finding is that c-Fos expression in the CA3 region varied depending on the subfield analyzed. Similar to the hilus, differential activation was observed in CA3c and, to a very similar extent, in CA3b, suggesting that these regions are similarly and progressively involved in pattern separation processes. Studies indicate that distinct subfields along the transverse axis of CA3 may serve specialized functions (Maurer et al., 2017b; Berdugo-Vega et al., 2023). The CA3c subfield is more directly connected to the hilus and the granule cell layer via mossy fibers, making it particularly relevant for minimizing memory overlap through pattern separation—a critical operation for memory encoding and retrieval (Senzai and Buzsáki, 2017; Senzai, 2018; Lee et al., 2022). Entorhinal inputs target the DG differentially, while projections from ventral CA1 and ventral subiculum preferentially innervate CA3a rather than CA3b/c. In contrast, CA3b/c receives more input from the perirhinal cortex. Within the DG, the supra- and infrapyramidal blades project preferentially to CA3ab and CA3c, respectively. Altogether, these anatomical and functional patterns support the idea of parallel processes along the CA3 axis. Whereas CA3c activity aligns with pattern separation computations, activity in CA3ab appears to be progressively linked to pattern completion (Berdugo-Vega et al., 2023; Maurer et al., 2017b; Witter, 2007; Lee et al., 2022, Lee et al., 2020).

We found strong positive correlations in CA3b and CA3c, but not in the hilus, indicating that CA3 activity is more directly related to discrimination performance. This can be explained by the dense recurrent excitatory connections in CA3, which allow this region to efficiently perform pattern completion and separation processes, making it particularly sensitive to differences in input patterns during memory tasks (Senzai, 2018; Lee et al., 2022; Berdugo-Vega et al., 2023). In addition, anatomical and computational evidence supports the notion that CA3 integrates convergent inputs from both the dentate gyrus (DG) and the entorhinal cortex (EC) to sustain memory encoding and retrieval (Yassa and Stark, 2011). In contrast, the hilus is primarily composed of mossy cells and interneurons, which modulate overall network excitability and information flow rather than directly encoding discrimination-related activity (GoodSmith et al., 2019; Scharfman, 2016). Therefore, CA3 activity tends to reflect the more detailed discrimination processes measured by our behavioral indices (Senzai, 2018).

No differences in c-Fos expression were observed between groups with higher similarity levels in the rostral hippocampus. This suggests that even as similarity increases, c-Fos expression in CA3 remains at a stable level, implying that a balanced activation level may be optimal for discrimination. Studies suggest an inverted U-shaped relationship between CA3 activity and the ability to discriminate between stimuli that share features (Cooper et al., 2022). Discrimination accuracy declines if CA3 activity is too low or too high (Kent et al., 2016; Lee et al., 2021; Maurer et al., 2017b). Duffy et al. (2013) showed an inhibitory relationship between the Hilus (with higher activation) and the granule cell layer (with lower activation). The lack of differential expression in the granule cell layer may be explained by the physiological properties of granule cells, which are typically more silent and hyperpolarized (Chawla et al., 2005; Jung and McNaughton, 1993).

Although functional differences have been proposed for activated cells in the DG along the dorsoventral axis in a longitudinal plane (Fredes et al., 2021; Houser et al., 2021), in our study, we did not perform direct comparisons of activated cells between the hippocampal subregion (rostral, medial and caudal). Instead, we focused on exploring the specific cell activation within the experimental groups for each subregion. Thus, the absence of differences along the anteroposterior axis in our study may be attributed to the fact that we divided the hippocampal formation into more subregions than usual in order to better explore the connectivity within each one under specific condition, as well as subdividing the caudal region in dorsal and ventral subareas. Additionally, this may be related to the specific nature of the task, which could be further explored in future studies by our lab and others. Unlike paradigms such as contextual fear conditioning, which strongly engages ventral hippocampal circuits associated with emotional processing (see Fanselow and Dong, 2010 for review), our task involved object recognition, a cognitive paradigm predominantly linked to perceptual discrimination and mnemonic processing, which are more closely associated with dorsal and rostral hippocampal regions, as found in our study to be dependent on the level of similarity.

Regarding the parahippocampal regions, it is noteworthy that a difference in c-Fos expression was observed only in the Prh36*dl* region. This area has been identified as central to the pattern separation process and is considered particularly specialized in object recognition, receiving various associative projections (Burke et al., 2018; Ahn and Lee, 2017; Pereira et al., 2016). Here, the increased c-Fos expression occurs only in the 50% similarity condition, suggesting that this region operates with an activation level similar to CA3c and the Hilus. In this context, the Prh36*dl* may be interacting with DG pathways to support the successful storage of similar objects (Miranda et al., 2021).

Several studies have indicated that hyperactivity in regions of the medial temporal lobe (MTL) may be linked to deficits in pattern separation processes. Therefore, an optimal level of activation must be maintained to disambiguate and effectively resolve the task (Maurer et al., 2017a; Ahn and Lee, 2017; Frick et al., 2023). Additionally, studies have shown that object recognition recruits immediate-early gene expression in layer V of areas 35 and 36 (Burke et al., 2012). Thus, the deep region of area 36 in the perirhinal cortex may play a key role in decoding object features. The absence of differences between the low-similarity condition and the controls may be explained by the nature of the objects used in the task (LEGOs), which can be considered complex and ambiguous, increasing the task’s difficulty. Consequently, the NOR control condition might be very similar to the low-similarity condition (Aggleton et al., 2010; Gámiz and Gallo, 2012).

The c-Fos connectome may reflect specific brain networks developed for memory processing. Here, network strength, as well as local and global efficiency, increased with higher similarity levels, except in the 75% condition. This finding mirrors results from spatiotemporal interference tasks, in which greater spatial interference is associated with more robust, efficient, and interconnected networks (Castelo-Branco et al., 2025). This suggests that conditions involving greater similarity—and therefore greater disambiguation demands—require greater efficiency in the global flow of information between regions, at global levels.

Furthermore, the DIST condition, with different levels of interference occurring simultaneously, required greater network efficiency, likely due to the constantly changing level of demand. As a result, the networks involved during the 25%, DIST, and 50% conditions may become progressively more plastic and robust, allowing a balanced integration of local and global processing, ultimately leading to highly efficient information transfer at low energy costs (Durieux et al., 2022). This would be essential for effective pattern separation and distinct storage of overlapping inputs, given that electrophysiological recordings, IEG studies, and high-resolution fMRI in humans have demonstrated that the dynamics of pattern separation/completion in DG and CA3 are complex and largely dependent on the degree of input similarity (Yassa and Stark, 2011). The NOR network, in turn, presented higher clustering values, suggesting that for this task the network can work with better communication between nearby nodes, revealing local communication (Fornito et al., 2016).

Higher similarity conditions exhibited lower modularity values, indicating greater network homogeneity, especially in the 50 and 75% conditions. This finding is in line with the notion that higher similarity requires a more integrated network to solve the task (Yassa and Stark, 2011). Furthermore, the way in which clusters connect appears to recruit hippocampal regions that are functionally interconnected through direct projections and are markedly distinct between conditions. For example, in the 75% condition, all rostral CA3 subregions were recruited along with other medial CA3 areas, replicating anatomically established projections (Berdugo-Vega et al., 2023).

The lack of an increase in network efficiency between the 50 and 75% conditions may be due to the absence of a measure to assess the “quality” of memory retrieval in high similarity conditions, which may be more challenging and therefore influence information storage. In human studies, ROC curves are commonly used as a measure of response confidence (Clark and Squire, 1998). Furthermore, intrinsic motivational or attentional factors also influence pattern separation processes (Senzai and Buzsáki, 2017).

When examining c-Fos expression in interneurons, distinct structures along the hippocampal axis showed significant differences, reflecting robust interneuron activity under high-similarity conditions within the DG-CA3 circuit. The inhibitory network may be acting to orthogonalize sensory inputs by inhibiting non-essential neurons and selecting key neurons for non-overlapping information storage—thus supporting pattern separation under high-similarity conditions (Madar et al., 2019). The increased activation of inhibitory neurons in the Hilus may indicate local inhibition of mossy cells (MCs), a finding later confirmed by SEM analysis (Larimer and Strowbridge, 2008). Differences in c-Fos expression among interneurons occurred similarly across nearly all granule cell layers (GCLs), except for the caudal dorsal layer, suggesting possible direct inhibition of granule cells. Given that dorsal Hilus projections predominantly target the middle molecular layer along the dorsoventral hippocampal axis—precisely where GABAergic interneurons from the GCL extend most of their dendrites—it is plausible that increased hilar activity in the dorsal hippocampus facilitates functional modulation and recruitment of these interneurons throughout the DG (Houser et al., 2021), which align with recent findings showing dendritic inhibition as an important mechanism of GC excitability and recruitment of interneuron activity on boundaries of granular layer (Elgueta and Bartos, 2019), which affect the sparse representation by granular cells and synaptic plasticity within DG.

When evaluating c-Fos+ activated/PV+inhibitory activated network connectivity in the DG-CA3 microcircuit, it becomes evident that inhibition mediated by PV-expressing interneurons is not static but dynamically adjusts across experimental conditions. This reflects the circuit’s functional plasticity and its adaptive responses to varying demands (Rolls, 2013; Espinoza et al., 2018; Hainmueller et al., 2024). Hippocampal segments were differentially engaged and inhibited depending on similarity levels. Such reorganization may reflect compensatory strategies and adaptive heterogeneity in maintaining excitatory-inhibitory balance. This aligns with expectations, as pattern separation requires differential recruitment of inhibitory neurons, which must operate distinctly across the hippocampus, confirming the structural and functional heterogeneity of this region (Botterill et al., 2021). One perspective posits that longitudinal connections originating from the Hilus constitute a broad inhibitory system that restricts granule cell activity across much of the DG, allowing for selective granule cell activation (Zappone and Sloviter, 2004; Jinde et al., 2012; Sloviter and Lømo, 2012). The functional role of these longitudinal hilar connections likely depends on the net effects of their projections and is thus modulated by varying conditions (Houser et al., 2021).

The previous finding is confirmed when we examine the generated model. The comparison between the constrained and unconstrained versions of the model with indirect paths, based on the DIC difference (>10 points), is interpreted as substantial evidence supporting the superiority of the more flexible model (Spiegelhalter et al., 2002). This reinforces the hypothesis that the mechanisms modulating granule cell activation are not uniform across experimental groups but instead are shaped by specific features of each condition (Rolls, 2013; Espinoza et al., 2018).

The results suggest that the functional engagement of the dentate gyrus microcircuit is minimal in conditions involving low object similarity: NOR, DIST, and 25%. As stimulus similarity increases (50, 75%), excitatory and inhibitory circuits progressively modulate granule layer activity in a more complex manner. This pattern aligns with the literature showing that pattern separation in the hippocampus occurs only when required (Leutgeb et al., 2007; Yassa and Stark, 2011). This supports the notion that the dentate gyrus becomes more actively engaged when stimuli are partially similar, requiring greater discrimination. The simultaneous involvement of multiple significant pathways in these conditions strengthens the hypothesis that pattern separation relies on specific interactions between inhibitory and excitatory neuronal populations, particularly under ambiguous encoding demands (Rolls, 2013). The fact that beta coefficients are not significantly different across groups, yet only functionally significant in certain ones, points to a task-dependent functional activation effect.

The 50% group, representing the intermediate similarity condition, exhibited the highest functional integration between layers and PV interneurons, with significant indirect effects, indicating a more robust recruitment of mechanisms involved in pattern separation. Although the 75% condition featured greater stimulus similarity, only the 50% condition demonstrated significance across all pathways. In the 75% condition, the positive direct and negative indirect pathways from hilar PV interneurons to granule cells were not significant, indicating a reduced influence of this interneuron population on granule cells under higher similarity conditions. However, direct inhibitory pathways from PV interneurons to mossy cells, and all other mechanisms modulating mossy cells’ influence on granule cells, remained active. This result is consistent with the view that pattern separation in the dentate gyrus is a gradual and adaptive process rather than binary or maximized at all costs (Yassa and Stark, 2011). Computational studies have shown that finer separations require greater inhibitory recruitment and increased synaptic thresholds (Chavlis and Poirazi, 2017). Thus, the reduced significance observed in the 75% group may reflect a functional or adaptive network limit in the face of high discrimination demands. Given that behavioral performance was similar across these conditions, this finding suggests that the network may achieve sufficient separation to support behavior even without full engagement of all mechanisms. Alternatively, the network may be influenced by other factors previously mentioned, such as response quality, attention, and motivational aspects (Senzai and Buzsáki, 2017).

Our results suggest that c-Fos+ activated cells (perhaps mossy cells, a major cell type within the Hilus in the DG) may exert dual control over granule cells: a direct excitatory pathway promoting specific activation of neuronal subpopulations, and an indirect inhibitory pathway suppressing collateral activation. Studies show that mossy fiber activation generates excitatory postsynaptic potentials (EPSPs) in granule cells, while also enhancing lateral inhibition via interneurons (Scharfman, 2016). This dynamic balance (evidenced by the non-significant total effect) may represent a key mechanism for pattern separation, ensuring that similar inputs produce distinct neural representations without saturating the circuit, through dual control over granule cells (Guzman et al., 2021; Chavlis and Poirazi, 2017). These findings support the role of the dentate gyrus as a “noise filter” for the formation of precise memories. Simulations have shown that the excitation-inhibition balance in the dentate gyrus is critical for generating sparse, non-overlapping representations (Chavlis and Poirazi, 2017).

Although an important methodological limitation of this study is the interpretation that the c-Fos-positive and PV-non-reactive cells could be excitatory neurons in the DG, it is noteworthy to mention the possible involvement of mossy cells (MC) in the DG interconnectivity in our model. This interpretation is further supported by studies reporting a significant subset—close to 100%—of c-Fos+ cells are MCs in the Hilus of rats exposed to either contextual (Duffy et al., 2013) or emotional (Moretto et al., 2017) stimuli. Additionally, another study reported that approximately 10–15% of the c-Fos+ cells in the Hilus are GABAergic neurons (Bernstein et al., 2019) aligning with earlier studies suggesting that about 20% of the population in the entire DG are GABAergic (Nomura et al., 1997). Moreover, other quantitative anatomical studies have suggested that the GABAergic neuron population in the DG is relatively low and also diverse. For example, in the granular cell layer, GABAergic neurons represent only about 2% of the total cell population (Woodson et al., 1989). In the hilus, on the other hand, approximately 36% of neurons are GABA-immunoreactive subdivided in several subtypes, including many that do not express parvalbumin (PV). While our findings cannot entirely rule the involvement of the broader GABergic population, our interpretation focused specifically on PV-expressing cells distributed throughout the DG and their connectivity along the transverse axis of the hippocampus has found support in recent studies highlighting the unique role of PV+ interneurons within the DG, especially at the border of the Hilus and granule cell layer (Jinno et al., 2006), where these cells express both GAD-65 and GAD-67 enzymes (Wang et al., 2014), exhibit distinctive electrophysiological properties, providing ten times more lateral inhibition than recurrent inhibition in granule cells (Espinoza et al., 2018), through both chemical and electrical synapses, and play a protective role against neuronal deterioration including under neurological conditions.

Another important limitation to consider is that we modeled the connectivity between activated neurons and/or PV-activated neurons in the multiple-trials task using only male rats, which compromises and limits generalization of our findings to females. Many authors have investigated the role of different neuronal types in the granule cell layer and hilus across the dentate gyrus; however, to our knowledge, most of these studies have focused on male subjects or have not provided clear sex differences. In this regard, we acknowledge that important questions remain open and deserve further investigation, including how the sex hormones interact within the dentate gyrus and affect behavioral strategies. Furthermore, the mechanisms observed in our findings likely reflect a combination of encoding and retrieval processes, which could be better disentangled in future investigations using both sexes and employ techniques with higher temporal resolution, such as electrophysiological studies. It is essential for future research to explore what occurs under non-discrimination conditions, enhancing our understanding of the underlying processes. The hippocampal inhibitory network is complex, and further studies should investigate how different types of interneurons contribute to pattern separation. From these observations, important theoretical questions arise about which inputs modulate inhibition related to stimulus similarity. Projections from the medial and lateral entorhinal cortex and the CA3 feedback circuit are key candidates to play a central role in this inhibitory process. However, the extent and manner in which these inputs interact and coordinate their actions remain open questions. These findings could be expanded through protocols involving optogenetic manipulations or electrical stimulation to modulate inputs and interactions within this circuit. Furthermore, using animal models that mimic aging and pathological conditions such as epilepsy and neurodegenerative diseases could help identify disruptions in hippocampal and cortical networks underlying failures in pattern separation.

Ultimately, the findings presented in this study help to clarify the neurobiological mechanisms involved in the recognition and disambiguation of objects with varying levels of similarity. Alongside previous results, it is demonstrated here that not only do DG-CA3 layers function differently to resolve high-similarity conditions, but an entire functionally engaged, heterogeneous hippocampal–parahippocampal network is robustly recruited to solve such tasks. Excitatory and inhibitory repercussions occur not only at the network level but also within the microcircuit layers of the dentate gyrus, modulating interactions between its inhibitory and excitatory cell types, with direct effects on granule cells. The proposed model demonstrates that excitatory and inhibitory modulation within the dentate gyrus (DG) is dependent on stimulus similarity, with significant recruitment occurring in conditions of higher similarity. This detailed analysis revealed the excitatory and inhibitory microcircuits involved in object recognition memory and pattern separation—processes particularly vulnerable to morphological and functional changes associated with aging and neurodegenerative diseases.

## Data Availability

The raw data supporting the conclusions of this article will be made available by the authors, without undue reservation.
