# Supplementary material for: Between similarity and difference: network dynamics of the hippocampal- parahippocampal circuitry in pattern separation of male Wistar rats
Source: Front Cell Neurosci. 2025 Nov 18;19:1648536. doi: 10.3389/fncel.2025.1648536 (PMC12669181; doi:10.3389/fncel.2025.1648536)
Supplement: Supplementary file 1 [file Table_1.docx]

**Supplementary Table 1.** Discrimination index (D2) for each experimental condition.

|  | Discrimination index (D2) | | | | | | | |
| --- | --- | --- | --- | --- | --- | --- | --- | --- |
| Condition | trials | t |  | p |  | Mean |  | Standard error |
| NOR | 1 | 3,227 |  | 0,015* |  | 0,338 |  | 0,104 |
|  | 2 | 2,698 |  | 0,031* |  | 0,312 |  | 0,115 |
|  | 3 | 3,192 |  | 0,015* |  | 0,300 |  | 0,094 |
|  | 4 | 3,215 |  | 0,015* |  | 0,238 |  | 0,074 |
| DIST | 1 | 0,545 |  | 0,602 |  | 0,038 |  | 0,071 |
|  | 2 | 1,366 |  | 0,214 |  | 0,157 |  | 0,115 |
|  | 3 | 2,802 |  | 0,026* |  | 0,255 |  | 0,091 |
|  | 4 | 2,633 |  | 0,034* |  | 0,240 |  | 0,091 |
| 25% | 1 | 1,067 |  | 0,335 |  | 0,072 |  | 0,068 |
|  | 2 | 2,000 |  | 0,102 |  | 0,109 |  | 0,054 |
|  | 3 | 1,155 |  | 0,300 |  | 0,074 |  | 0,064 |
|  | 4 | 3,732 |  | 0,014* |  | 0,104 |  | 0,028 |
| 50% | 1 | -0,199 |  | 0,848 |  | -0,017 |  | 0,086 |
|  | 2 | 1,789 |  | 0,124 |  | 0,150 |  | 0,083 |
|  | 3 | 2,487 |  | 0,047* |  | 0,165 |  | 0,066 |
|  | 4 | 3,019 |  | 0,023* |  | 0,151 |  | 0,050 |
| 75% | 1 | 3,946 |  | 0,011* |  | 0,308 |  | 0,078 |
|  | 2 | 1,677 |  | 0,154 |  | 0,206 |  | 0,123 |
|  | 3 | 2,712 |  | 0,042* |  | 0,227 |  | 0,083 |
|  | 4 | 2,806 |  | 0,038* |  | 0,208 |  | 0,074 |

* Significant at the p<0,05 level.

**Supplementary Table 2.** Results of control statistical analyses (mixed-effects ANOVA).

| **Variable / Comparison** | **Effect tested** |  | **F (df1, df2)** |  | **p-value** |
| --- | --- | --- | --- | --- | --- |
| ***D2*** | Condition |  | F(4, 29) = 0.58 |  | 0.679 |
|  | Trial |  | F(3, 87) = 0.25 |  | 0.864 |
|  | Condition × Trial |  | F(12, 87) = 0.70 |  | 0.747 |
| ***Exploration time (sample phase)*** | Object (within sample) |  | F(1, 31) = 0.34 |  | 0.562 |
|  | Obj × Condition |  | F(4, 40) = 0.38 |  | 0.818 |
|  | Trial |  | F(2.66, 82.5) = 0.07 |  | 0.968 |
|  | Trial × Condition |  | F(10.36, 106.7) = 1.56 |  | 0.211 |
|  | Condition (between-subjects) |  | F(4, 31) = 0.61 |  | 0.660 |

| **Supplementary Table 3** – Statistical comparisons of c-Fos expression in each hippocampal area across all conditions. | | | |
| --- | --- | --- | --- |
| **Hippocampal Portion** | **Test statistics** | | |
|  | ***Área*** | ***F ou H*** | ***p value*** |
| ***Rostral*** |  |  |  |
|  | CA3a | H=9.067 | 0.059 |
|  | CA3b | H=12.496 | 0.014* |
|  | CA3c | H=4.677 | 0.005* |
|  | GCL | H=4.094 | 0.393 |
|  | Hilus | F= 4.514 | 0.006* |
| ***Medial*** |  |  |  |
|  | CA3a | F=0.491 | 0.742 |
|  | CA3b | H=2.093 | 0.0719 |
|  | CA3c | H=4.690 | 0.321 |
|  | GCL | F=1.194 | 0.335 |
|  | Hilus | F= 2.920 | 0.571 |
| ***Caudal (Dorsal)*** |  |  |  |
|  | CA3*d* | H=4.877 | 0.3 |
|  | GCL*d* | H=2.085 | 0.72 |
|  | Hilus*d* | F=0.515 | 0.725 |
| ***Caudal (Ventral)*** |  |  |  |
|  | CA3*v* | H=0.802 | 0.938 |
|  | GCL*v* | H=3.656 | 0.301 |
|  | Hilus*v* | F=1.397 | 0.28 |
| ***Cortex*** |  |  |  |
|  | Prh36s*l* | F=2,605 | 0,061 |
|  | Prh36*dl* | H=10,260 | 0,036* |
|  | Prh35*sl* | F=0,763 | 0,560 |
|  | Prh35*dl* | H=2,908 | 0,573 |
|  | LEC*dl* | H=1,599 | 0,809 |
|  | LECs*l* | F=1,776 | 0,167 |
| *Significant at the p<0,05 level. | | | |
| *F= One Way ANOVA, H= Kruskal-Wallis Test. | | | |

|  | **Supplementary Table 4** - Planned contrasts with activated c-Fos in each area between conditions. | | | | |
| --- | --- | --- | --- | --- | --- |
|  | **Hippocampal portion** | **Area** | **Experimental conditions** | **Test statistics** | |
|  |  |  |  | **t value** | **p value** |
|  | ***Rostral*** | **CA3c** | NOR and DIST x 25%, 50% e 75% | 2.844 | 0.008* |
|  |  |  | 25% x 50% e 75% | 2.996 | 0.006* |
|  |  |  | 50% x 75% | -1.085 | 0.287 |
|  |  |  | NOR x DIST | 0.405 | 0.688 |
|  |  | **Hilus** |  |  |  |
|  |  |  | NOR and DIST x 25%, 50% e 75% | 2.871 | 0.008* |
|  |  |  | 25% x 50% e 75% | 2.753 | 0.010* |
|  |  |  | 50% x 75% | -1.706 | 0.099 |
|  |  |  | NOR x DIST | -0.545 | 0.59 |
|  | ***Medial*** | **CA3a** |  |  |  |
|  |  |  | NOR and DIST x 25%, 50% e 75% | 1.286 | 0.209 |
|  |  |  | 25% x 50% e 75% | 0.278 | 0.783 |
|  |  |  | 50% x 75% | -0.414 | 0.682 |
|  |  |  | NOR x DIST | -0.402 | 0.691 |
|  |  | **GCL** |  |  |  |
|  |  |  | NOR and DIST x 25%, 50% e 75% | 0.929 | 0.361 |
|  |  |  | 25% x 50% e 75% | 1.04 | 0.307 |
|  |  |  | 50% x 75% | -1.511 | 0.142 |
|  |  |  | NOR x DIST | 0.374 | 0.711 |
|  | *Significant at the p<0,05 level. | | | | |

| **Supplementary Table 5** - Graph centrality measures of all c-fos+ for each region in each condition, Part 1. | | | | | | | | | |
| --- | --- | --- | --- | --- | --- | --- | --- | --- | --- |
| **Areas and *Hippocampus portion*** | **NOR** | | | **DIST** | | | **25%** | | |
|  | **Degree** | **Centrality** | **Strength** | **Degree** | **Centrality** | **Strength** | **Degree** | **Centrality** | **Strength** |
| ***Rostral*** |  |  |  |  |  |  |  |  |  |
| CA3a | 1* | 0* | 0.840* | 10* | 22.13 | 8.636* | 0* | 0* | 0* |
| CA3b | 2 | 0* | 1.697 | 5 | 5.683* | 4.355 | 3* | 0* | 2.423* |
| CA3c | 3* | 48* | 2.654* | 2* | 0* | 1.664* | 5* | 20.63 | 4.084 |
| GCL | 1* | 0* | 0.942* | 4* | 19.87* | 3.153* | 1* | 0* | 0.857* |
| Hilus | 0* | 0* | 0* | 8* | 74.61* | 6.873* | 6* | 5.366* | 5.624* |
| ***Medial*** |  |  |  |  |  |  |  |  |  |
| Ca3a | 3* | 90* | 2.711* | 10* | 26.21* | 8.540* | 10* | 30.08* | 9.049* |
| CA3b | 3 | 0* | 2.739* | 9* | 7.461* | 7.493* | 10* | 36.36 | 9.149* |
| CA3c | 4* | 65.5* | 3.549* | 6 | 25.32* | 5.204 | 11* | 50.78* | 9.899* |
| GCL | 5* | 34* | 4.483* | 6 | 27.73* | 5.247 | 4 | 2.5* | 3.518 |
| Hilus | 2 | 0* | 1.723 | 12* | 65.20* | 10.41* | 6* | 5.366* | 5.660* |
| ***Caudal (Dorsal)*** |  |  |  |  |  |  |  |  |  |
| CA3*d* | 3* | 5.5* | 2.771* | 1* | 0* | 0.942* | 0* | 0* | 0* |
| GCL*d* | 1* | 0* | 0.885* | 1* | 0* | 0.892* | 3* | 0.833* | 2.726* |
| Hilus*d* | - | - | - | 4* | 32.24* | 3.239* | 6* | 27.54 | 5.539* |
|  |  |  |  |  |  |  |  |  |  |
| ***Caudal (Ventral)*** |  |  |  |  |  |  |  |  |  |
| CA3*v* | 5* | 20.5* | 4.510* | 1* | 0* | 0.942* | 0* | 0* | 0* |
| GCL*v* | 0* | 0* | 0* | 6 | 26.54* | 5.142 | 0* | 0* | 0* |
| Hilus*v* | - | - | - | 6* | 63.68* | 5.032* | 0* | 0* | 0* |
| *Significant at the p<0,05 level. | | | | | | | | | |

| **Supplementary Table 6** - Graph centrality measures of all c-fos+ for each region in each condition, Part 2. | | | | | | |
| --- | --- | --- | --- | --- | --- | --- |
| **Areas and *Hippocampus portion*** | **50%** | | | **70%** | | |
|  | **Degree** | **Centrality** | **Strength** | **Degree** | **Centrality** | **Strength** |
| ***Rostral*** |  |  |  |  |  |  |
| CA3a | 18* | 21.77* | 16.09* | 2* | 8* | 1.783* |
| CA3b | 15* | 9.216* | 13.36* | 5* | 5.330* | 4.799* |
| CA3c | 15* | 8.549* | 13.28* | 4* | 0* | 3.567* |
| GCL | 9* | 8.109* | 7.382 | 6* | 4.896* | 5.612* |
| Hilus | 2* | 0* | 1.678* | 0* | 0* | 0* |
| ***Medial*** |  |  |  |  |  |  |
| CA3a | 12* | 3.351* | 10.74* | 1* | 0* | 0.811* |
| CA3b | 8 | 4.963* | 6.790* | 5* | 2.5* | 4.654* |
| CA3c | 14* | 7.685* | 12.26* | 8* | 57.81* | 7.090* |
| GCL | 12* | 1.666* | 10.60* | 8* | 26.45* | 7.555* |
| Hilus | 13* | 18.47 | 11.47* | 4 | 19* | 3.553 |
| ***Caudal (Dorsal)*** |  |  |  |  |  |  |
| CA3*d* | 2* | 0* | 1.885* | 0* | 0* | 0* |
| GCL*d* | 12* | 2.920* | 10.68* | 6* | 4.896* | 5.612* |
| Hilus*d* | 17* | 13.20* | 14.82* | 3 | 10* | 2.640* |
| ***Caudal (Ventral)*** |  |  |  |  |  |  |
| CA3*v* | 0* | 0* | 0* | 0* | 0* | 0* |
| GCL*v* | 0* | 0* | 0* | 3* | 0* | 2.974 |
| Hilus*v* | 4* | 0* | 3.974* | 0* | 0* | 0* |
| *Significant at the p<0,05 level. | | | | | | |

| **Supplementary Table 7** - Network efficiency measures including all c-Fos+ cells | | | | |
| --- | --- | --- | --- | --- |
| Conditions | **Global Efficiency** | | **Cluster coefficient** | |
|  | Original value | van Wijk normalization | Original value | van Wijk normalization |
| NOR | 0.225 | 0.71** | 0.505 | 7.89** |
| DIST | 0.512 | 0.92** | 0.606 | 2.79** |
| 25% | 0.388 | 0.72** | 0.524 | 3.42** |
| 50% | 0.457 | 0.74** | 0.719 | 2.24** |
| 75% | 0.262 | 0.62** | 0.611 | 3.50** |
| **Represents the values ​​of statistical tests whose significance was p<0.05 level in bootstrap and permutation test. | | | | |

**Supplementary Table 8** - Louvain cluster analysis values, modules and modularity for network with all c-Fos+

| **Group** | **Hippocampus Portion** | **Group Number** | **Areas** | **Isolated** |
| --- | --- | --- | --- | --- |
| **NOR** | ***Rostral*** | 4 | CA3a, CA3b, CA3c |  |
|  |  | 5 | GCL |  |
|  |  | Isolated |  |  |
|  | ***Medial*** | 3 | CA3c, Hilus | Hilus |
|  |  | Isolated |  | GCL |
|  | ***Medial*** | 8 | CA3a, CA3b |  |
|  | ***Caudal dorsal*** | 3 | CA3*d* |  |
|  |  | Isolated |  | Hilus*d* |
|  | ***Caudal dorsal*** | 5 | GCL*d* |  |
|  | ***Caudal ventral*** | 3 | CA3*v* |  |
| **DIST** | ***Rostral*** | 1 | CA3a, CA3b, CA3c |  |
|  | ***Rostral*** | 2 | GCL, Hilus |  |
|  | ***Medial*** | 1 | CA3a, CA3b, Hilus |  |
|  | ***Medial*** | 2 | GCL |  |
|  | ***Medial*** | 4 | CA3c |  |
|  | ***Caudal dorsal*** | 2 | GCL*d*, Hilus*d* |  |
|  | ***Caudal dorsal*** | 5 | CA3*d* |  |
|  | ***Caudal ventral*** | 2 | Hilus*v* |  |
|  | ***Caudal ventral*** | 4 | GCL*v* |  |
|  | ***Caudal ventral*** | 5 | CA3*v* |  |
| **25%** | ***Rostral*** | 1 | Hilus |  |
|  | ***Rostral*** | 4 | CA3b |  |
|  | ***Rostral*** | 6 | CA3c |  |
|  | ***Rostral*** | Isolated |  | CA3a |
|  | ***Medial*** | 1 | CA3b, Hilus |  |
|  | ***Medial*** | 4 | CA3a, CA3c |  |
|  | ***Medial*** | 6 | GCL |  |
|  | ***Caudal dorsal*** | 1 | Hilus*d* |  |
|  | ***Caudal dorsal*** | 4 | GCL*d* |  |
|  | ***Caudal dorsal*** | 6 | GCL*d* |  |
|  | ***Caudal dorsal*** | Isolated |  | CA3*d* |
|  | ***Caudal ventral*** | Isolated |  | CA3*v*, GCL *v*, Hilus*v* |
| **50%** | ***Rostral*** | 5 | CA3c, Hilus, GCL |  |
|  | ***Rostral*** | 6 | CA3a, CA3b |  |
|  | ***Medial*** | 1 | CA3b |  |
|  | ***Medial*** | 5 | GCL |  |
|  | ***Medial*** | 6 | CA3a, CA3c, Hilus |  |
|  | ***Caudal dorsal*** | 1 | Hilus*d* |  |
|  | ***Caudal dorsal*** | 5 | GCL*d* |  |
|  | ***Caudal dorsal*** | 6 | CA3*d* |  |
|  | ***Caudal ventral*** | 6 | Hilus*v* |  |
|  | ***Caudal ventral*** | Isolated |  | CA3*v*, GCL*v* |
| **75%** | ***Rostral*** | 1 | GCL |  |
|  | ***Rostral*** | 7 | CA3a, CA3b, CA3c |  |
|  | ***Rostral*** | Isolated |  | Hilus |
|  | ***Medial*** | 1 | GCL |  |
|  | ***Medial*** | 2 | CA3a, Hilus |  |
|  | ***Medial*** | 7 | CA3b, CA3c |  |
|  | ***Caudal dorsal*** | 1 | GCL*d* |  |
|  | ***Caudal dorsal*** | 7 | Hilus*d* |  |
|  | ***Caudal dorsal*** | Isolated |  | CA3*d* |
|  | ***Caudal ventral*** | 2 | GCL*v* |  |
|  | ***Caudal ventral*** | Isolated |  | CA3*v* ,Hilus *v* |

| **Supplementary Table 9** - Statistical comparison of modularity of network with all c-fos between condition | | | | | |
| --- | --- | --- | --- | --- | --- |
| Measures | **Modularity value (**van Wijk normalization**)** | | | | |
|  | NOR | DIST | 25% | 50% | 75% |
| Louvain modularity | 0.612 (1.22)** | 0.034 (1.38)** | 0.385 (1.15)** | 0.037 (0.75)** | 0.429 (1.08)** |
| **Represents the values ​​of statistical tests whose significance was p<0.05 level in bootstrap and permutation test. | | | | | |

| **Supplementary Table 10** – Statistical comparisons of activated interneurons (PV/c-Fos) in each hippocampal area across all conditions. | | | |
| --- | --- | --- | --- |
| **Hippocampus portion** | **Test statistics**** | | |
|  | ***Área*** | ***F ou H*** | ***p value*** |
| ***Rostral*** |  |  |  |
|  | CA3a | H=8.032 | 0.09 |
|  | CA3b | H=11.982 | 0.017* |
|  | CA3c | H=13.378 | 0.010* |
|  | GCL | F=3.954 | 0.011* |
|  | Hilus | H=12.484 | 0.014* |
| ***Medial*** |  |  |  |
|  | CA3a | H=12.068 | 0.017* |
|  | CA3b | F=1.747 | 0.168 |
|  | CA3c | H=11.984 | 0.017* |
|  | GCL | F=4.770 | 0.005* |
|  | Hilus | H=15.344 | 0.004* |
| ***Caudal (Dorsal)*** |  |  |  |
|  | CA3*d* | 10.656 | 0.031* |
|  | GCL*d* | 8.278 | 0.082 |
|  | Hilus*d* | 20.074 | 0.001* |
| ***Caudal (Ventral)*** |  |  |  |
|  | CA3*v* | 5.707 | 0.222 |
|  | GCL*v* | 3.490 | 0.039* |
|  | Hilus*v* | 2.213 | 0.126 |
| *Significant at the p<0,05 level. | | | |
| *F= One Way ANOVA, H= Kruskal-Wallis Test. | | | |

| **Supplementary Table 11** - Planned contrasts with activated interneurons (PV/c-Fos) in each area between conditions. | | | |
| --- | --- | --- | --- |
| ***Hippocampus portion* and areas** | **Experimental conditions** | **Test statistics** | |
|  |  | **t value** | **p value** |
| ***Rostral*** | | | |
| GCL | NORe DIST x 25%, 50% e 75% | 2.46 | 0.02* |
|  | 25% x 50% e 75% | 3.33 | 0.007* |
|  | 50% x 75% | -0.384 | 0.710 |
|  | NOR x DIST | -2.168 | 0.057 |
| ***Medial*** |  |  |  |
| CA3b | NORe DIST x 25%, 50% e 75% | 0.863 | 0.396 |
|  | 25% x 50% e 75% | 1.623 | 0.116 |
|  | 50% x 75% | -1.674 | 0.105 |
|  | NOR x DIST | -1.127 | 0.269 |
| GCL |  |  |  |
|  | NORe DIST x 25%, 50% e 75% | 2.99 | 0.007* |
|  | 25% x 50% e 75% | 3.70 | 0.03* |
|  | 50% x 75% | -1.881 | 0.070 |
|  | NOR x DIST | -0.622 | 0.539 |
| ***Caudal (Dorsal)*** |  |  |  |
| GCL*d* | NORe DIST x 25%, 50% e 75% | 1.850 | 0.075 |
|  | 25% x 50% e 75% | 1.804 | 0.082 |
|  | 50% x 75% | -1.222 | 0.232 |
|  | NOR x DIST | 0.817 | 0.421 |
|  |  |  |  |
| ***Caudal (Ventral)*** |  |  |  |
| GCL*v* |  |  |  |
|  | DIST x 25%, 50% e 75% | 2.073 | 0.054 |
|  | 25% x 50% e 75% | 2.106 | 0.050 |
|  | 50% x 75% | 0.377 | 0.711 |
| Hilus*v* |  |  |  |
|  | DIST x 25%, 50% e 75% | 1.326 | 0.203 |
|  | 25% x 50% e 75% | 1.602 | 0.129 |
|  | 50% x 75% | -1.267 | 0.223 |
| *Significant at the p<0,05 level. | | | |

| **Supplemental Table 12** - Graph centrality measures of c-Fos+ and PV+/c-Fos+ cells, for each region in each condition, Part 1. | | | | | | | | | | |
| --- | --- | --- | --- | --- | --- | --- | --- | --- | --- | --- |
| **Cell type** | ***Hippocampus portion* and areas** | **NOR** | | | **DIST** | | | **25%** | | |
|  |  | **Degree** | **Centrality** | **Strength** | **Degree** | **Centrality** | **Strength** | **Degree** | **Centrality** | **Strength** |
| ***Excitatory*** | ***Rostral*** |  |  |  |  |  |  |  |  |  |
| ***cell*** | CA3a | 3* | 13.5* | 2.652* | 5 | 30.333* | 4.136* | 3* | 7* | 2.653* |
|  | CA3b | 3* | 13.5* | 2.652* | 2 | 4* | 1.654 | 0* | 0* | 0* |
|  | CA3c | 2 | 2* | 1.705 | 1* | 0* | 0.811* | 3* | 3.333* | 2.655* |
|  | GCL | 0* | 0* | 0.000 | 1* | 0* | 0.896* | 2* | 0* | 1.911* |
|  | Hilus | 2 | 0* | 1.813 | 4* | 10* | 3.366* | 0* | 0* | 0* |
|  |  |  |  |  |  |  |  |  |  |  |
|  | ***Medial*** |  |  |  |  |  |  |  |  |  |
|  | CA3a | 1* | 0* | 0.880* | 5* | 2* | 4.152* | 4* | 10* | 3.526* |
|  | CA3b | 4* | 40 | 3.594* | 0* | 0* | 0* | 3* | 5.333* | 2.674* |
|  | CA3c | 2 | 0* | 1.635 | 4* | 1.333* | 3.383* | 2* | 0* | 1.788* |
|  | GCL | 1* | 0* | 0.853* | 3* | 22* | 2.525* | 1* | 0* | 0.943* |
|  | Hilus | 2 | 2* | 1.739 | 4* | 30.333* | 3.347* | 3* | 3.333* | 2.716* |
|  |  |  |  |  |  |  |  |  |  |  |
|  | ***Caudal (Dorsal)*** |  |  |  |  |  |  |  |  |  |
|  | CA3*d* | 2 | 0* | 1.784 | 0* | 0* | 0* | 0* | 0* | 0* |
|  | GCL*d* | 4 | 5.5* | 3.711* | 1* | 0* | 0.954* | 1* | 0* | 0.928* |
|  | Hilus*d* | 0* | 0* | 0* | 1* | 0* | 0.937* | 1 | 0* | 0.812 |
|  |  |  |  |  |  |  |  |  |  |  |
|  | ***Caudal (Ventral)*** |  |  |  |  |  |  |  |  |  |
|  | CA3*v* | 2 | 12* | 1.739 | 0* | 0* | 0* | 0* | 0* | 0* |
|  | GCL*v* | - | - | - | 2 | 0* | 1.812 | 0* | 0* | 0* |
|  | Hilus*v* | - | - | - | 1* | 0* | 0.767* | 0* | 0* | 0* |
| ***Inhibitory*** | ***Rostral*** |  |  |  |  |  |  |  |  |  |
| ***cell*** | CA3a | 1* | 0* | 0.880* | 2 | 3* | 1.834 | 2* | 0* | 1.865* |
|  | CA3b | 4* | 52* | 3.276* | 1* | 0* | 0.896* | 6* | 27* | 5.096* |
|  | CA3c | 1* | 0* | 0.820* | 4* | 22* | 3.300* | 0* | 0* | 0* |
|  | GCL | 2 | 0* | 1.826 | 3* | 12* | 2.399 | 0* | 0* | 0* |
|  | Hilus | 1* | 0* | 0.899* | 2 | 3* | 1.656 | 0* | 0* | 0* |
|  |  |  |  |  |  |  |  |  |  |  |
|  | ***Medial*** |  |  |  |  |  |  |  |  |  |
|  | CA3a | 1* | 0* | 0.868* | 0* | 0* | 0* | 1 | 0* | 0.813* |
|  | CA3b | 2 | 12* | 1.750 | 0* | 0* | 0* | 0* | 0* | 0* |
|  | CA3c | 4* | 5.5* | 3.722* | 0* | 0* | 0* | 0* | 0* | 0* |
|  | GCL | 4* | 12* | 3.722* | 1* | 0* | 0.807* | 3* | 9* | 2.617* |
|  | Hilus | 3* | 22* | 2.564 | 1* | 0* | 0.881* | 2* | 0* | 1.925* |
|  |  |  |  |  |  |  |  |  |  |  |
|  | ***Caudal (Dorsal)*** |  |  |  |  |  |  |  |  |  |
|  | CA3*d* | 0* | 0* | 0* | 3* | 3* | 2.569* | 1 | 0* | 0.895 |
|  | GCL*d* | 1* | 0* | 0.853* | 3* | 3* | 2.572* | 2 | 1* | 1.739 |
|  | Hilus*d* | 0* | 0* | 0* | 2 | 0* | 1.838 | 3* | 7* | 2.709* |
|  |  |  |  |  |  |  |  |  |  |  |
|  | ***Caudal (Ventral)*** |  |  |  |  |  |  |  |  |  |
|  | CA3*v* | 0* | 0* | 0* | 2 | 0* | 1.813 | 1* | 0* | 0.895* |
|  | GCL*v* | - | - | - | 6* | 14* | 5.083* | 0* | 0* | 0* |
|  | Hilus*v* | - | - | - | 2 | 0* | 1.799 | 0* | 0* | 0* |
| *Significant at the p<0,05 level. | | | | | | | | | | |

| **Supplementary Table 13** - Graph centrality measures of c-Fos+ and PV+/c-Fos+ cells, for each region in each condition, Part 2. | | | | | | | |
| --- | --- | --- | --- | --- | --- | --- | --- |
| **Cell type** | **Areas and *Hippocampus portion*** | **50%** | | | **70%** | | |
|  |  | **Degree** | **Centrality** | **Strength** | **Degree** | **Centrality** | **Strength** |
| ***Excitatory*** | ***Rostral*** |  |  |  |  |  |  |
| ***cell*** | CA3a | 2* | 45* | 1.714* | 1 | 0* | 0.812* |
|  | CA3b | 2 | 4* | 1.571* | 1* | 0* | 0.886* |
|  | CA3c | 3* | 0* | 2.529* | 1* | 0* | 0.820* |
|  | GCL | 4* | 0* | 3.447* | 1* | 0* | 0.841* |
|  | Hilus | 1* | 0* | 0.883* | 0* | 0* | 0* |
|  |  |  |  |  |  |  |  |
|  | ***Medial*** |  |  |  |  |  |  |
|  | CA3a | 1* | 0* | 0.893* | 3 | 30 | 2.800* |
|  | CA3b | 2* | 3* | 1.679* | 2 | 0* | 1.793 |
|  | CA3c | 3 | 87* | 2.714 | 2 | 0* | 1.623 |
|  | GCL | 0* | 0* | 0* | 2* | 2* | 1.697 |
|  | Hilus | 1* | 0* | 0.786* | 2 | 40* | 1.798 |
|  |  |  |  |  |  |  |  |
|  | ***Caudal (Dorsal)*** |  |  |  |  |  |  |
|  | CA3*d* | 0* | 0* | 0* | 0* | 0* | 0* |
|  | GCL*d* | 3 | 80* | 2.464 | 0* | 0* | 0* |
|  | Hilus*d* | 2 | 17 | 1.571 | 3* | 22 | 2.737* |
|  |  |  |  |  |  |  |  |
|  | ***Caudal (Ventral)*** |  |  |  |  |  |  |
|  | CA3*v* | 0* | 0* | 0* | 0* | 0* | 0* |
|  | GCL*v* | 2 | 81* | 2 | 0* | 0* | 0* |
|  | Hilus*v* | 0* | 0* | 0* | 0* | 0* | 0* |
| ***Inhibitory*** | ***Rostral*** |  |  |  |  |  |  |
| ***cell*** | CA3a | 3* | 32 | 2.562* | 3* | 0.5* | 2.730* |
|  | CA3b | 1* | 0* | 0.883* | 0* | 0* | 0* |
|  | CA3c | 5* | 0.667* | 4.03* | 0* | 0* | 0* |
|  | GCL | 4* | 0* | 3.447* | 3* | 22 | 2.670* |
|  | Hilos | 2* | 3* | 1.75* | 3* | 22 | 2.791* |
|  |  |  |  |  |  |  |  |
|  | ***Medial*** |  |  |  |  |  |  |
|  | CA3a | 5* | 4* | 4.205* | 2* | 12 | 1.639 |
|  | CA3b | 1* | 0* | 0.893* | 0* | 0* | 0* |
|  | CA3c | 8* | 18.833* | 6.820* | 4* | 3.5* | 3.569* |
|  | GCL | 8* | 18.833* | 6.757* | 2* | 2* | 1.652* |
|  | Hilus | 1* | 0* | 0.964* | 2 | 36* | 1.751 |
|  |  |  |  |  |  |  |  |
|  | ***Caudal (Dorsal)*** |  |  |  |  |  |  |
|  | CA3*d* | 2 | 0* | 1.652 | 2 | 0* | 1.921* |
|  | GCL*d* | 4* | 0* | 3.474* | 2 | 0* | 1.756 |
|  | Hilus*d* | 2 | 0* | 1.734 | 2 | 30 | 1.797 |
|  |  |  |  |  |  |  |  |
|  | ***Caudal (Ventral)*** |  |  |  |  |  |  |
|  | CA3*v* | 0* | 0* | 0* | 2 | 0* | 1.842* |
|  | GCL*v* | 6* | 80.667* | 5.541* | 3* | 42* | 2.924* |
|  | Hilus*v* | 0* | 0* | 0* | 2* | 0* | 1.922* |
| *Significant at the p<0,05 level. | | | | | | | |

| **Supplementary Table 14** – Measures for c-Fos+ and PV+/c-Fos+ cells network efficiency | | | | |
| --- | --- | --- | --- | --- |
| Conditions | **Global Efficiency** | | **Cluster coefficient** | |
|  | Original value | van Wijk normalization | Original value | van Wijk normalization |
| NOR | 0.153 | 0.251** | 0.510 | 0.074** |
| DIST | 0.149 | 0.293** | 0.500 | 0.081** |
| 25% | 0.087 | 0.134** | 0.409 | 0.047** |
| 50% | 0.185 | 0.355** | 0.605 | 0.102** |
| 75% | 0.111 | 0.164** | 0.417 | 0.054** |
| **Represents the values ​​of statistical tests whose significance was p<0.05 level in bootstrap and permutation test. | | | | |

| **Supplementary Table 15** - Table with statistical comparison of modularity for c-Fos+ and PV+/c-Fos+ cells between condition | | | | | |
| --- | --- | --- | --- | --- | --- |
| Measures | **Modularity value (**van Wijk normalization**)** | | | | |
|  | NOR | DIST | 25% | 50% | 75% |
| Louvain modularity | 0.666 (0.561)** | 0.643 (0.538)** | 0.534 (0.624)** | 0.579 (0.484)** | 0.703 (0.618)** |
| **Represents the values ​​of statistical tests whose significance was p<0.05 level in bootstrap and permutation test. | | | | | |

| **Supplementary Table 16 -** Condition fit indices for two theoretical models: direct paths and direct paths plus indirect paths | | | | | |
| --- | --- | --- | --- | --- | --- |
| ***Model*** | ***Conditions*** | ***Model fit indices*** | | | |
|  |  | **PPP** | **DIC** | **WAIC** | **LOOIC** |
| Direct + indirect regressions Model | 25% | 0,378 | 36,305 | 41,554 | 45,537 |
| Direct + indirect regressions Model | 50% | 0,456 | 31,751 | 39,48 | 41,69 |
| Direct + indirect regressions Model | 75% | 0,482 | 36,353 | 43,045 | 44,398 |
| Direct + indirect regressions Model | DIST | 0,289 | 24,662 | 32,097 | 35,143 |
| Direct + indirect regressions Model | NOR | 0,082 | 31,771 | 37,268 | 38,044 |
| Direct regressions Model | 25% | 0,544 | 20,43 | 22,21 | 24,086 |
| Direct regressions Model | 50% | 0,384 | -10,301 | -2,344 | -0,466 |
| Direct regressions Model | D75 | 0,397 | 4,148 | 11,482 | 12,739 |
| Direct regressions Model | DIST | 0,411 | 4,534 | 8,66 | 10,255 |
| Direct regressions Model | NOR | 0,402 | 5,974 | 11,684 | 12,749 |
| *Significant at the p<0,05 level. | | | | | |

| **Supplementary Table 17 -** Parameters of each regression of the theoretical model with indirect paths by condition | | | | |
| --- | --- | --- | --- | --- |
| **Conditions** | **Parameter** | **Beta** | **Lower IC** | **Upper IC** |
| ***NOR*** | GC ← MC | 686 | -303 | 1.084 |
|  | GC ← PV in GCL | 27 | -937 | 774 |
|  | GC ← PV in Hilus | 389 | -109 | 852 |
|  | PV in GCL ← MC | 258 | -0.07 | 644 |
|  | MC ← PV in Hilus | -313 | -659 | -37 |
|  | GC ~~ GC | 106 | 4 | 533 |
|  | PV in GCL ~~ PV in GCL | 904 | 585 | 999 |
|  | MC ~~ MC | 875 | 566 | 998 |
|  | PV in Hilus ~~ PV in Hilus | 1.0 | 1.0 | 1.0 |
|  | Indirect effect MC | 2 | -358 | 222 |
|  | Indirect effect PV in Hilus | -218 | -643 | 77 |
|  | Total effect MC to GC | 687 | -526 | 1.099 |
|  | Total effect PV in Hilus to GC | 171 | -226 | 585 |
| ***DIST*** | GC ← MC | 508 | -852 | 1.473 |
|  | GC ← PV in GCL | -561 | -1.093 | 414 |
|  | GC ← PV in Hilus | 408 | -971 | 1.389 |
|  | PV in GCL ← MC | 519 | 0.07 | 829 |
|  | MC ← PV in Hilus | -699 | -945 | -253 |
|  | GC ~~ GC | 352 | 46 | 884 |
|  | PV in GCL ~~ PV in GCL | 691 | 313 | 988 |
|  | MC ~~ MC | 476 | 107 | 936 |
|  | PV in Hilus ~~ PV in Hilus | 1.0 | 1.0 | 1.0 |
|  | Indirect effect MC | -298 | -809 | 231 |
|  | Indirect effect PV in Hilus | -359 | -1.276 | 624 |
|  | Total effect MC to GC | 0.21 | -0.94 | 1.181 |
|  | Total effect PV in Hilus to GC | 49 | -596 | 604 |
| ***25%*** | GC ← MC | 0.27 | -462 | 0.9 |
|  | GC ← PV in GCL | -203 | -976 | 773 |
|  | GC ← PV in Hilus | 52 | -512 | 596 |
|  | PV in GCL ← MC | 326 | -223 | 774 |
|  | MC ← PV in Hilus | -153 | -356 | -9 |
|  | GC ~~ GC | 403 | 49 | 897 |
|  | PV in GCL ~~ PV in GCL | 832 | 391 | 999 |
|  | MC ~~ MC | 968 | 873 | 1.0 |
|  | PV in Hilus ~~ PV in Hilus | 1.0 | 1.0 | 1.0 |
|  | Indirect effect MC | -67 | -0.55 | 351 |
|  | Indirect effect PV in Hilus | -39 | -172 | 65 |
|  | Total effect MC to GC | 203 | -684 | 902 |
|  | Total effect PV in Hilus to GC | 13 | -545 | 538 |
| ***50%*** | GC ← MC | 553 | 162 | 1.096 |
|  | GC ← PV in GCL | -1.048 | -1.334 | -829 |
|  | GC ← PV in Hilus | 374 | 68 | 675 |
|  | PV in GCL ← MC | 488 | 136 | 817 |
|  | MC ← PV in Hilus | -0.54 | -857 | -144 |
|  | GC ~~ GC | 37 | 2 | 219 |
|  | PV in GCL ~~ PV in GCL | 0.73 | 332 | 981 |
|  | MC ~~ MC | 671 | 265 | 979 |
|  | PV in Hilus ~~ PV in Hilus | 1.0 | 1.0 | 1.0 |
|  | Indirect effect MC to GC | -527 | -1.073 | -127 |
|  | Indirect effect PV in Hilus | -287 | -613 | -52 |
|  | Total effect MC to GC | 27 | -406 | 495 |
|  | Total effect PV in Hilus to GC | 87 | -167 | 362 |
| ***75%*** | GC ← MC | 673 | 58 | 1.135 |
|  | GC ← PV in GCL | -736 | -961 | -0.21 |
|  | GC ← PV in Hilus | 452 | -104 | 809 |
|  | PV in GCL ← MC | 197 | 31 | 0.47 |
|  | MC ← PV in Hilus | -0.47 | -784 | -79 |
|  | GC ~~ GC | 106 | 4 | 633 |
|  | PV in GCL ~~ PV in GCL | 948 | 779 | 999 |
|  | MC ~~ MC | 742 | 386 | 992 |
|  | PV in Hilus ~~ PV in Hilus | 1.0 | 1.0 | 1.0 |
|  | Indirect effect MC | -0.14 | -331 | -7 |
|  | Indirect effect PV in Hilus | -317 | -729 | 13 |
|  | Total effect MC to GC | 533 | -32 | 0.95 |
|  | Total effect PV in Hilus to GC | 135 | -328 | 515 |
| *Significant at the p<0,05 level. | | | | |

## **Table 18- Raw PV+/Cfos+ expression in rostral hippocampus**

| **CA3a** | **CA3b** | **CA3c** | **Granular** | **Hilus** | **Condition** |
| --- | --- | --- | --- | --- | --- |
| 0 | 0 | 0 | 6 | 3 | NOR |
| 1 | 0,5 | 0,5 | 1 | 2 | NOR |
| 1,5 | 2 | 2 | 6,5 | 8,5 | NOR |
| 2 | 2 | 0 | 5 | 3 | NOR |
| 2 | 1,5 | 0 | 2,5 | 5,5 | NOR |
| 2 | 1 | 1 | 2,5 | 4 | NOR |
| 1 | 0 | 0 | 0 | 2 | 25% |
| 0,5 | 0 | 0 | 2,5 | 3 | 25% |
| 0 | 1 | 0 | 1 | 3 | 25% |
| 2,5 | 2 | 0 | 0,5 | 1,5 | 25% |
| 0,5 | 0 | 0 | 1 | 0 | 25% |
| 0,5 | 0 | 0 | 2 | 2 | 25% |
| 0 | 0 | 0 | 3 | 2,5 | 25% |
| 1 | 1,3 | 0 | 1,3 | 1,3 | 50% |
| 1,5 | 1 | 3 | 2,5 | 5,5 | 50% |
| 2 | 0 | 0 | 2 | 23 | 50% |
| 0 | 1,5 | 3,5 | 12 | 6,5 | 50% |
| 5 | 3,5 | 3 | 7 | 9 | 50% |
| 5,5 | 1,5 | 4 | 8 | 10 | 50% |
| 4 | 3 | 3 | 10 | 3 | 50% |
| 0 | 0 | 0 | 1,5 | 0 | 75% |
| 2,5 | 1 | 1,5 | 0,5 | 9 | 75% |
| 3 | 2,5 | 0 | 0,5 | 8,5 | 75% |
| 4,5 | 2 | 0,5 | 8,5 | 7,5 | 75% |
| 2 | 4 | 3 | 12 | 12 | 75% |
| 2,5 | 5,5 | 5 | 12 | 8,5 | 75% |
| 2 | 0 | 0 | 0 | 19 | DIST |
| 2,5 | 0,5 | 1,5 | 3,5 | 6 | DIST |
| 0 | 0 | 1 | 0,5 | 4,5 | DIST |
| 0 | 1 | 0,5 | 2 | 2,5 | DIST |
| 2 | 0 | 0 | 2,5 | 3 | DIST |
| 1,5 | 0 | 0 | 0,5 | 1,5 | DIST |
| 0 | 0,5 | 0,5 | 1 | 3 | DIST |

##

## **Table 19- Raw PV+/Cfos+ expression in medial hippocampus**

| **CA3a** | **CA3b** | **CA3c** | **Granular** | **Hilus** | **Condition** |
| --- | --- | --- | --- | --- | --- |
| 2,5 | 2 | 4 | 4 | 2,5 | NOR |
| 2 | 1 | 0,5 | 1,5 | 3,5 | NOR |
| 3 | 1 | 0 | 0 | 2 | NOR |
| 1 | 0 | 0 | 0 | 1 | NOR |
| 2 | 0,5 | 1 | 1,5 | 1 | NOR |
| 10,5 | 2,5 | 8,5 | 7,5 | 11,5 | NOR |
| 0 | 0 | 0 | 2 | 2 | 25% |
| 0,5 | 0 | 0 | 1 | 1 | 25% |
| 2 | 1 | 1 | 2 | 3 | 25% |
| 2,5 | 0,5 | 0 | 2,5 | 0,5 | 25% |
| 0,5 | 0 | 0,5 | 0,5 | 1,5 | 25% |
| 2 | 1,5 | 1 | 2 | 1 | 25% |
| 6 | 1,5 | 0 | 2 | 4 | 25% |
| 2 | 1,5 | 1 | 1 | 1,5 | 50% |
| 10 | 3,5 | 2 | 5,5 | 9 | 50% |
| 2 | 0 | 0 | 2 | 23 | 50% |
| 2,5 | 0,5 | 2,5 | 7 | 10 | 50% |
| 2,5 | 1,5 | 1,5 | 4 | 12,5 | 50% |
| 10,5 | 2,5 | 8,5 | 7,5 | 11,5 | 50% |
| 4 | 3 | 3 | 10 | 3 | 50% |
| 4 | 0 | 5 | 2 | 6 | 75% |
| 10 | 3 | 2 | 2 | 12 | 75% |
| 4 | 0,5 | 1,5 | 3 | 9 | 75% |
| 2 | 1 | 0 | 1 | 4 | 75% |
| 3 | 1 | 5 | 8 | 12 | 75% |
| 4 | 0 | 3 | 6 | 5 | 75% |
| 2 | 0 | 0 | 0 | 19 | DIST |
| 1 | 0,5 | 0 | 3 | 5 | DIST |
| 3 | 1,5 | 0,5 | 1,5 | 3,5 | DIST |
| 1,5 | 1 | 0 | 1 | 1,5 | DIST |
| 2 | 1 | 0,5 | 2,5 | 1 | DIST |
| 1 | 0,5 | 0 | 1 | 1 | DIST |
| 0,5 | 0 | 0,5 | 1,5 | 1,5 | DIST |

## **Table 20- Raw PV+/Cfos+ expression in caudal hippocampus**

| **CA3*d*** | **CA3*v*** | **Granular*d*** | **Granular*v*** | **Hilus*d*** | **Hilus*v*** | **Condition** |
| --- | --- | --- | --- | --- | --- | --- |
| 4 | 2 | 1 | N/A | 3,5 | N/A | NOR |
| 3 | 4 | 1 | 3 | 4 | 6 | NOR |
| 7,5 | 6 | 3,5 | N/A | 6 | N/A | NOR |
| 2 | 1 | 2 | N/A | 2 | N/A | NOR |
| 0 | 4 | 2 | N/A | 3 | N/A | NOR |
| 4 | 4 | 4 | N/A | 3 | N/A | NOR |
| 4 | 7 | 1 | 0 | 2 | 0 | 25% |
| 0 | 0 | 1 | 0 | 6 | 2 | 25% |
| 0 | 0 | 7 | 4,5 | 12 | 12,5 | 25% |
| 0 | 0 | 2 | 2 | 2 | 1 | 25% |
| 5 | 4 | 3 | 0 | 3 | 0 | 25% |
| 1 | 0,5 | 4 | 1 | 2,5 | 1 | 25% |
| 3 | 5 | 2 | 0 | 7 | 0 | 50% |
| 8 | 2 | 5 | 6 | 10 | 5 | 50% |
| 8 | 5 | 2 | 1 | 18 | 5 | 50% |
| 2 | 0 | 4 | 4,5 | 8 | 18 | 50% |
| 11 | 8 | 5 | 2 | 28 | 19 | 50% |
| N/A | N/A | 10 | 11 | 27 | 46 | 50% |
| 7 | 7 | 8 | 10 | 17 | 0 | 50% |
| 3 | 4 | 4 | 5 | 10 | 17 | 75% |
| 5,5 | 6 | 2,5 | 8 | 18 | 9,5 | 75% |
| 5 | 6 | 2 | 10 | 15 | 7 | 75% |
| N/A | N/A | 4 | 4 | 7 | 7 | 75% |
| 17 | 10 | 4 | 0 | 17 | 0 | 75% |
| 5 | 5 | 8,5 | 5 | 14,5 | 15,5 | 75% |
| 2 | 2 | 2 | 0 | 6 | 1 | DIST |
| 5,5 | 5,5 | 5 | 3,5 | 13 | 9 | DIST |
| 5,5 | 8,5 | 5,5 | 4,5 | 10 | 8 | DIST |
| 0 | 0 | 1 | 3 | 6 | 9 | DIST |
| 0 | 0 | 3,5 | 0 | 7 | 8 | DIST |
| 1 | 1 | 4 | 2 | 3 | 2 | DIST |
| 0 | 0 | 1 | 1 | 5 | 4 | DIST |





**Supplementary Figure 1.** Representative images of c-Fos and PV labeling in the dentate gyrus for each condition.


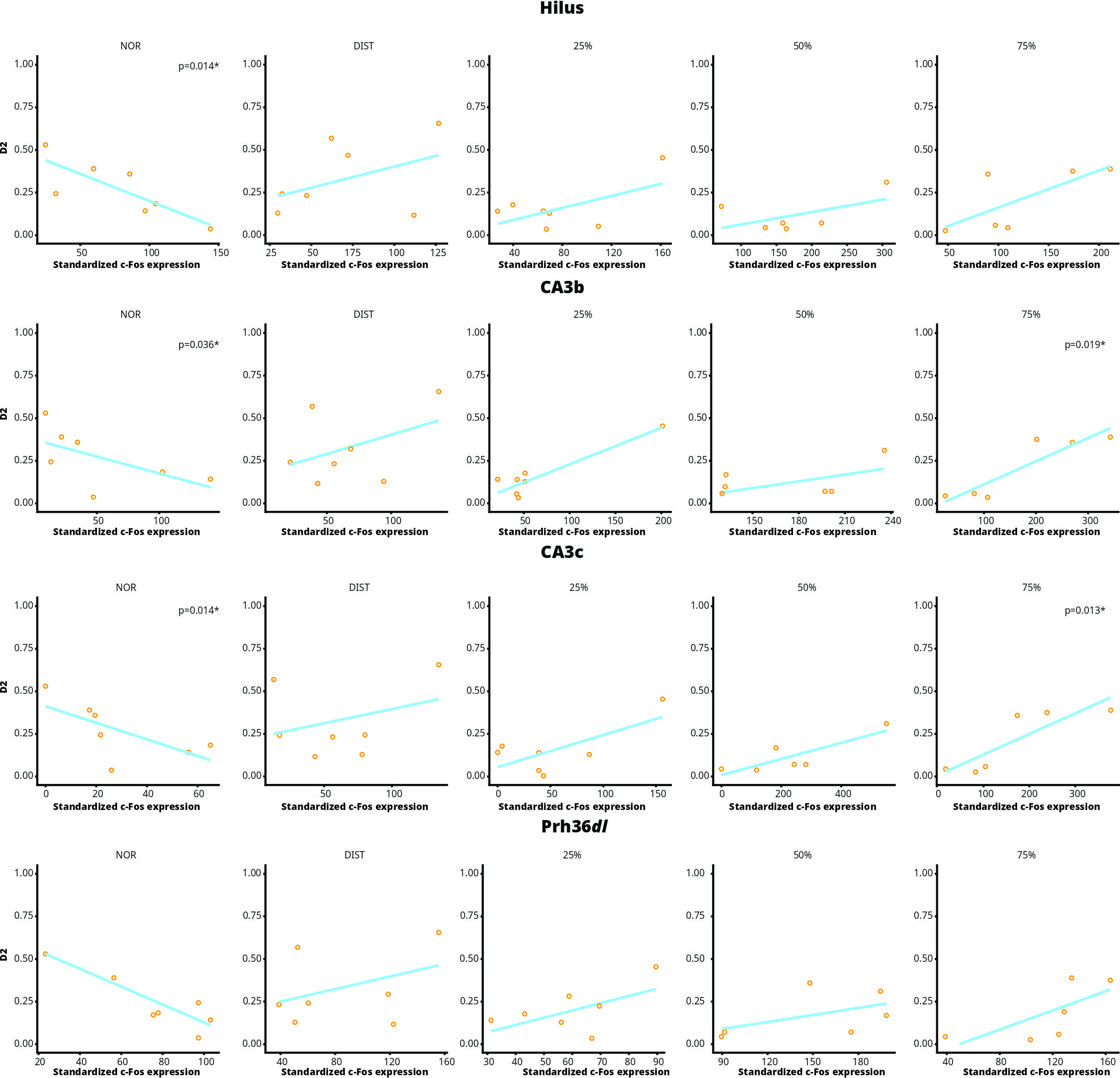


**Supplementary Figure 2.** Correlation plot between c-Fos expression and the cumulative discrimination index (4 trial) for each group in brain regions showing significant differences between groups.


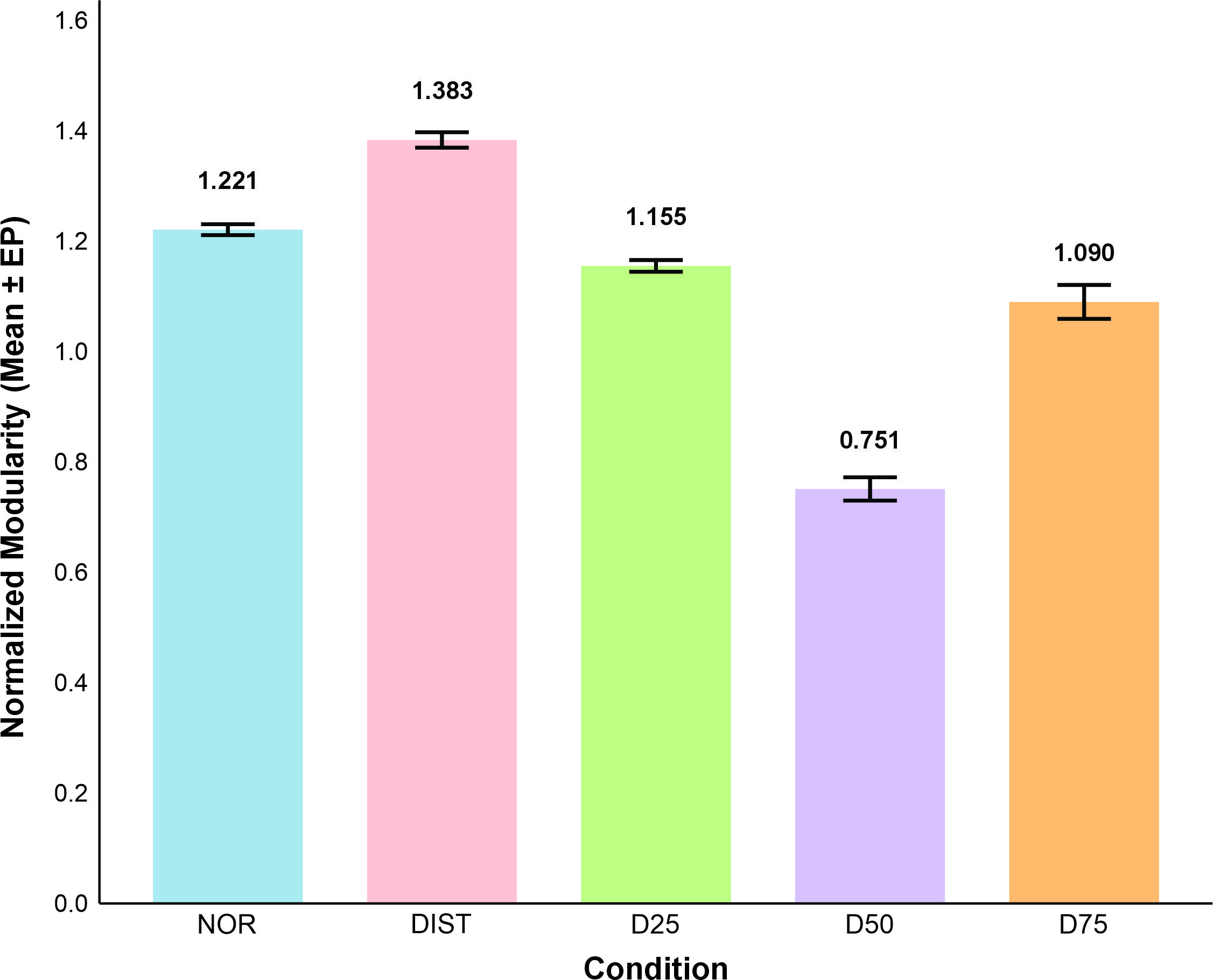


**Supplementary Figure 3.** Normalized Modularity of clusters for each condition; N=6-7. ± Standard Error of the mean.
